# KP372-1-Induced AKT Hyperactivation Blocks DNA Repair to Synergize With PARP Inhibitor Rucaparib *via* Inhibiting FOXO3a/GADD45α Pathway

**DOI:** 10.3389/fonc.2022.976292

**Published:** 2022-09-20

**Authors:** Lingxiang Jiang, Yingchun Liu, Xiaolin Su, Jiangwei Wang, Ye Zhao, Soumya Tumbath, Jessica A. Kilgore, Noelle S. Williams, Yaomin Chen, Xiaolei Wang, Marc S. Mendonca, Tao Lu, Yang-Xin Fu, Xiumei Huang

**Affiliations:** ^1^ Department of Radiation Oncology, Melvin and Bren Simon Comprehensive Cancer Center, Indiana University School of Medicine, Indianapolis, IN, United States; ^2^ Laboratory of Stem Cell Engineering and Regenerative Medicine, Fujian Province University/School of Basic Medical Sciences, Fujian Medical University, Fujian, China; ^3^ Departments of Biochemistry and Molecular Biology, Melvin and Bren Simon Comprehensive Cancer Center, Indiana University School of Medicine, Indianapolis, IN, United States; ^4^ Department of Biochemistry, Simmons Comprehensive Cancer Center, University of Texas (UT) Southwestern Medical Center, Dallas, TX, United States; ^5^ Indiana University Health Pathology Laboratory, Indiana University School of Medicine, Indianapolis, IN, United States; ^6^ State Key Laboratory of Applied Organic Chemistry, Department of Chemistry, Lanzhou University, Lanzhou, China; ^7^ Department of Pharmacology and Toxicology, Indiana University Melvin and Bren Simon Comprehensive Cancer Center, Indiana University School of Medicine, Indianapolis, IN, United States; ^8^ Department of Pathology, University of Texas Southwestern Medical Center, Dallas, TX, United States

**Keywords:** KP372-1, NQO1, AKT inhibitor, PARP inhibitor resistance, FOXO3a/GADD45α

## Abstract

Poly (ADP-ribose) polymerase (PARP) inhibitors (PARPi) have exhibited great promise in the treatment of tumors with homologous recombination (HR) deficiency, however, PARPi resistance, which ultimately recovers DNA repair and cell progress, has become an enormous clinical challenge. Recently, KP372-1 was identified as a novel potential anticancer agent that targeted the redox enzyme, NAD(P)H:quinone oxidoreductase 1 (NQO1), to induce extensive reactive oxygen species (ROS) generation that amplified DNA damage, leading to cancer cell death. To overcome PARPi resistance and expand its therapeutic utility, we investigated whether a combination therapy of a sublethal dose of KP372-1 with a nontoxic dose of PARPi rucaparib would synergize and enhance lethality in *NQO1* over-expressing cancers. We reported that the combination treatment of KP372-1 and rucaparib induced a transient and dramatic AKT hyperactivation that inhibited DNA repair by regulating FOXO3a/GADD45α pathway, which enhanced PARPi lethality and overcame PARPi resistance. We further found that PARP inhibition blocked KP372-1-induced PARP1 hyperactivation to reverse NAD^+^/ATP loss that promoted Ca^2+^-dependent autophagy and apoptosis. Moreover, pretreatment of cells with BAPTA-AM, a cytosolic Ca^2+^ chelator, dramatically rescued KP372-1- or combination treatment-induced lethality and significantly suppressed PAR formation and γH2AX activation. Finally, we demonstrated that this combination therapy enhanced accumulation of both agents in mouse tumor tissues and synergistically suppressed tumor growth in orthotopic pancreatic and non-small-cell lung cancer xenograft models. Together, our study provides novel preclinical evidence for new combination therapy in *NQO1^+^* solid tumors that may broaden the clinical utility of PARPi.

## Introduction

After the first promising clinical trials using a Poly (ADP-ribose) polymerase (PARP) inhibitor (PARPi) as treatments for platinum-sensitive *BRCA1/2* mutated breast and ovarian cancers, several PARP inhibitors have been approved by the FDA/EMA as monotherapies or combination therapies for *BRCA* mutated and/or platinum-sensitive breast and ovarian tumors (1, 2). Although the great promise of PARPi treatments in patients with homologous recombination (HR)-deficient tumors has been demonstrated, PARPi resistance has become a major clinical challenge (3, 4). Studies to date have revealed several mechanisms of PARPi resistance, all of which result in the restoration of DNA repair and in the resumption of cancer cell proliferation (3, 4). PARP is a family of nuclear enzymes mediating post-translational Poly(ADP-ribosyl)ation (PARylation) of substrate proteins involved in a number of cellular processes such as DNA damage repair, genomic stability, and programmed cell death (5). There are seventeen family members in the PARP family, however the PARP1 protein has been shown to play an important role in sensing DNA single strand breaks (SSBs) and double strand breaks (DSBs) (6, 7). When DNA strand breaks occur, PARP1 is activated and PARylates itself, creating a scaffold to recruit and activate central components of DNA damage checkpoint network including ataxia telangiectasia mutated kinase (ATM) by PARylation or stimulating DNA-dependent protein kinase (DNA-PK) that help facilitate DNA repair and cell survival (6, 8). In HR repair deficient (*BRCA1/2* deficient) tumors, which have deficient ability to restore the PARPi-induced DNA repair, PARP inhibitors block PARP activation and lead to cell death (3). However, PARP inactivation also results in the suppression of PARylation of ATM, and ATM, in turn, forms an ATM-NEMO complex that translocates to cytoplasm, where it activates AKT and subsequent cell survival pathways, leading to PARPi resistance (8, 9).

AKT, a serine/threonine-specific protein kinase with three isoforms: AKT1, 2, and 3, plays an essential role in phosphatidylinositol-3 kinase (PI3K) pathway that controls cell proliferation and pro-survival anti-apoptotic mechanisms (10). Dysregulation of the PI3K/AKT pathway is observed in many human cancers (11, 12), and in particular AKT has been found to be frequently activated in human cancers and is associated with poor prognosis and anticancer therapy resistance (13, 14). In PARPi treatments, activated AKT has been shown to contribute to drug resistance in cancer cells (9, 15). For example, exposure to alkylating agent MNNG and AG14361, a potential PARP1 inhibitor, was reported to significantly and durably increase phosphorylated AKT, leading to cancer cell growth recovery (16). Activated AKT regulates downstream substrates and participates in DNA repair, cell cycle arrest, drug efflux, and anti- apoptosis (17). In addition, inhibition of PI3K/AKT by PI3K or AKT inhibitors in *in vitro* or clinical trial has been shown to improve PARPi anticancer effects (18, 19). One proposed mechanism for the synergy between PARPi and AKT inhibitor is downregulated expression of HR components such as BRCA1 (19). Taken together, these studies indicate that PI3K/AKT pathway plays a pivotal role in the limitation of PARPi utilization in cancer treatment, and the potential utility of using AKT inhibition might overcome PARPi resistance and broaden PARPi clinical application.

NAD(P)H:quinone oxidoreductase (NQO1) is an obligate two-electron reductase that is involved in chemoprotection and can also bioactivate certain antitumor quinones (20). *NQO1* is overexpressed in most solid cancers (e.g., non-small cell lung, pancreatic, breast, and head and neck), with very low expression in normal cells/tissue (21, 22), and has the potential to be a promising therapeutic target for cancer treatment (23). KP372-1 (molecular structure shown in **Figure S1A**), was previously reported as a potent AKT inhibitor, shows evidence of single-agent activity to suppress AKT activity that inhibits cancer cells proliferation and induces apoptosis (24, 25). Recently, KP372-1 was also reported as a novel potential anticancer agent that targeted *NQO1* to induce extensive reactive oxygen species (ROS) generation that amplified DNA damage, leading to cancer cell death (26, 27). In addition, there are reports indicating that AKT hyperactivation promotes cell death and enhances the antitumor effects of chemotherapy in prostate and ovarian cancers *via* inhibiting forkhead box class O (FOXO) tumor suppressors and inducing reactive oxygen species (ROS), leading to cell senescence or ROS-induced apoptosis (28–30). Therefore, strategies to enhance KP372-1 efficacy without augmenting toxicity are needed. In 2020, Dr. Patidar research group revealed that the combination of KP372-1 with PARP inhibitor BMN 673 enhanced KP372-1-induced cytotoxicity in MiaPaCa-2 pancreatic cancer cells (27), however, the mechanism of this combination therapy remains unknown. We hypothesize that treatment with a PARPi rucaparib (FDA approved) prior to exposure to KP372-1 will enhance both drugs antitumor effects through KP372-1-induction of superoxidase and hyperactivation of AKT and PARPi’s inhibition of PARP-driven DNA repair in a tumor-selective manner and thereby overcome a major PARPi resistance mechanism.

## Materials and methods

### Drugs and reagents

KP372-1 was synthesized by Dr. Xiaolei Wang’s lab (Lanzhou University, China). Rucaparib was kindly provided by Clovis Oncology, Inc. Dicoumarol and Hoechst 33258 were purchased from Sigma-Aldrich. HPβCD (>98% purity) was obtained from Cyclodextrin Technologies Development, Inc. Antibodies used in this study for immunofluorescence and Western blotting were shown in Supplemental Material and Methods.

### Cell lines and cell culture

A549, MCF-7, MDA-MB-231, and MiaPaCa-2 were obtained from the American Tissue Culture Collection (ATCC, Manasas, VA). MDA-MB-231 *NQO1^+^* and MCF-7 sh*PARP1* were generated by us (21, 31). A549 and MiaPaCa-2 *NQO1^-^* cell lines were generated in our lab. Cells were grown as in Supplemental Material and Methods.

### *NQO1* and *PARP1* knocking out by CRISPR-Cas9 and siRNA transfection

Vectors of guide RNA sensing *NQO1* or non-target control (LV04) and Cas9 expression (CAS9NEO) were provided by Sigma-Aldrich, guide RNA targeting sequences are: AGGATACTGAAAGTTCGCAGGG, CACAATATCTGGGCTCAGATGG. Plasmid of *PARP1* CRISPR/Cas9 knockout (sc-400046) was obtained from Santa Cruz. More information about generating *NQO1* and *PARP1* knock-out cells or siRNA transfection was shown in Supplemental Material and Methods.

### Cell survival assays

Relative survival assays based on 7-day DNA content assessments were described as previous report (31). Colony formation assay were performed using 750 cells/6 cm plate. Colonies of > 50 healthy appearing cells were counted normalized to control cells.

### Western blotting

Westerns were performed using ECL chemiluminescent detection. Details were shown in Supplemental Material and Methods.

### ATP, H_2_O_2_ and NAD^+^ quantification

ATP (CellTiter-Glo^®^ 2.0), hydrogen peroxide (H_2_O_2_) (ROS-Glo™ H_2_O_2_), and NAD^+^ (NAD/NADH-Glo™) levels were assayed at 2 h after treatments according to the protocol (Promega, Madison, WI).

### Comet and immunofluorescence assays and immunohistochemistry staining

For comet assay, slides were stained with SYBR^®^ Gold TE solution and captured using a Leica DM5500 microscope. Comet tail lengths were quantified by NIH Image J. For γH2AX and RAD51 foci, drug-treated cells were immunofluorescence stained and imaged on a Leica DM5500 fluorescent microscope and quantified for foci/nucleus. For Immunohistochemistry staining, see the information in Supplemental Materia and Methods.

### Annexin-V FITC/7-AAD assay

Cells treated with drugs were harvested and washed with 1x PBS. 1 x 10^6^ cells were resuspended in 100 μL staining buffer and stained with both Annexin-V FITC and 7-AAD dye for 10 min according to manufacturer’s protocol. After that, 400 μL staining buffer was added to run flow cytometry. The apoptosis events were analyzed by FlowJo 10 software.

### O_2_ consumption rate assay

O_2_ consumption rate was measured using Seahorse 96-well plates in conjunction with an XF96 sensor cartridge and XF96 Extracellular Flux Analyzer (Agilent Technologies, DE) according to the manufacturer’s instructions.

### Antitumor efficacy and pharmacokinetic studies

Antitumor and survival were performed using orthotopic NSCLC A549 or pancreatic-specific MiaPaCa-2 xenograft-bearing NOD/SCID mice. Pharmacokinetic study was done using orthotopic NSCLC A549 xenograft-bearing NOD/SCID mice. All animal procedures were approved by the Indiana University IACUC committee. Bioluminescence (BLI)-based tumor volumes, long-term survival and target validation assays were performed with log-rank tests for survival. Pharmacokinetics (PK) of rucaparib or KP372-1 levels in blood, tumor, liver, and brain were assessed by LC-MS/MS analyses, following extraction of plasma or tissue homogenates with acetonitrile. More details on establishing orthotopic models and collecting samples for PK of rucaparib or KP372-1 assay were shown in Supplemental Material and Methods.

### Synergy calculations

**Figure 1:**
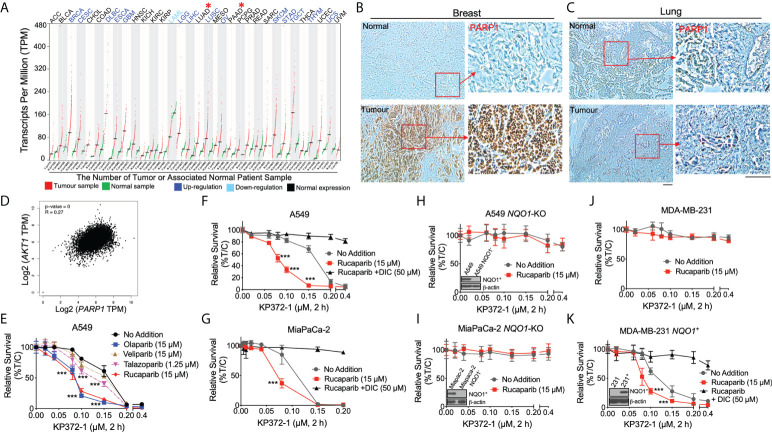
KP372-1 enhances the lethality of PARP inhibitors in various cancer cells and depends on *NQO1* activity. **(A)** *PARP1* mRNA expression in matched pan-cancer tumor tissue. Data were from TCGA and analyzed with GEPIA web server. Red color indicates tumor sample; green indicates associated normal patient sample; blue color suggests up-regulation of *PARP1*; light blue indicates down-regulation of *PARP1*; black indicates normal expression of *PARP1*. "*" (in red color) shows the representative cancer types we are focused of this article, which have normal PARP1 expression. **(B, C)** Representative IHC staining of PARP1 in breast **(B)** and lung **(C)** cancer patient samples or associated normal tissues. **(D)** Correlation between *AKT1* and *PARP1* in patient data obtained from TCGA. **(E)** Cell viability of combination treatment of KP372-1 with various PARP inhibitors in NSCLC A549 cells. **(F, G)** Cell viability of combination treatment of KP372-1 with rucaparib ± DIC in NSCLC A549 **(F)** and pancreatic cancer MiaPaCa-2 cells **(G)**. **(H, I)** Cell viability of combination treatment of KP372-1 ± rucaparib in *NQO1*-knockout A549 **(H)** and MiaPaCa-2 **(I)** cell lines. **(J, K)** Cell viability of combination treatment of KP372-1 ± rucaparib in MDA-MB-231 **(J)** and stable *NQO1* expressing MDA-MB-231 **(K)** cell lines. **(E–K)** Cells were pre-treated ± rucaparib or other PARP inhibitors for 2 h, then exposed to KP372-1 ± rucaparib or other PARP inhibitors for 2 h, followed by washing and replacing fresh media, Cell viability was determined by DNA assay 7 days later. Data are shown as mean ± SD, each experiment was done three independent times. Scale bar indicates 110 μm. **(E–G)** ***P < 0.001, comparing each data point with KP372-1 treatments (*t* tests).

Synergy interactions between the two drugs were evaluated using two methods (1): direct comparisons made between the effect of combined treatments and the effect of individual drugs in each experiment (**Figures 1E, F**, **3A**, **S1G–J, R**); and (2) formal synergy effects evaluations used a strict method proposed by Chou and coworkers (32, 33), where pooled, multiple dose responses for each treatments were required. Values (η) were reported based on multiple dose-response data from studies in **Figures 1E, F**, **3A**, **S1G, S1H, S1I, S1J** and **S1R**. We tested drug-drug interactions for three pairs: KP372-1 + rucaparib, KP372-1 + olaparib, and KP372-1 + talazoparib, all of which showed highly significant effect of synergy (η = 0.472, *P* = 0.004; η = 0.453, *P* = 0.002; η = 0.613, *P* = 0.009, respectively). For *in vivo* studies rucaparib + KP372-1 synergy showed an η value of 0.82, with *p* values indicated on graphs.

### Statistical analysis

The data were represented as mean ± SD from three independent experiments. Two-tailed Student’s *t*-tests for independent measures with Holm-Sidak correction for multiple comparisons, if > 1 comparisons, were performed. Statistical analyses were performed in GraphPad Prism 8 (GraphPad Software, Inc. CA, USA). Images were representative of results of experiments or staining repeated 3 times. *p* value of < 0.05 was considered as statistically significant between compared groups. *P < 0.05, **P < 0.01, and ***P < 0.001.

## Results

### KP372-1 enhances the lethality of PARP inhibitors in an *NQO1*-dependent manner

We examined *PARP1* mRNA expression in matched pan-cancer tumor tissues in the TCGA database and found that *PARP1* mRNA expression was elevated in 14 types of cancers (e. g., breast cancer (BRCA), ovarian (OV); blue color), and exhibited no significant change in 16 types of cancer (e.g., lung adenocarcinoma (LUAD), pancreatic cancer (PAAD); black color) compared to associated normal tissues (**Figure 1A**). We then examined clinical patient samples from our institution (Indiana University Melvin and Bren Simon Comprehensive Cancer Center Tissue Bank) and also demonstrated high PARP1 expression in breast cancer and low PARP1 expression in lung cancer (**Figures 1B, C** and **Figures S1B–E**). Additionally, the correlation analysis from the same TCGA pan-cancer data showed that *PARP1* expression strongly correlated with *AKT1* expression (**Figure 1D**). These findings together with previous reports (9, 34) suggest the possible efficacy of combining PARPi treatment with an AKT inhibitor in low PARP1 expressing solid cancers.

Our previous studies have revealed that the non-toxic dose (> 90% survival) of PARP inhibitors was 15 µM for rucaparib, olaparib and veliparib, and 1.25 µM for talazoparib in A549 NSCLC cells (21). To test the potential efficacy of combining PARPi with KP372-1, we examined the synergistic effect of various PARPi with KP372-1 in A549 NSCLC cells that have low PARP1 expression (**Figure S1F**). We found that treatment with the PARP inhibitors rucaparib and olaparib, each at 15 µM or talazoparib at 1.25 µM dramatically increased the sensitivities of A549 cells to sublethal doses of KP372-1 compared to 15 µM veliparib (**Figure 1E**). Dose-response studies for each PARP inhibitor confirmed that optimal synergistic lethality with KP372-1 was noted at 15 µM for rucaparib and olaparib, and at 1.25 µM for talazoparib. Veliparib was the least potent and effective PARP inhibitor for synergy with KP372-1 (**Figures S1G–J**). Synergy effects were calculated for KP372-1 + rucaparib, KP372-1 + olaparib and KP372-1 + talazoparib at eta (η) values of 0.472, 0.453 and 0.613, respectively (32, 33). Dicoumarol (DIC, an NQO1 specific inhibitor) prevented all synergy responses (**Figures S1G–J**). We chose rucaparib for further studies since clinical-grade formulation was available. Since the cancer toxicity of KP372-1 has been reported to have a potential dependence on NQO1 expression (26), we next examined the efficacy of combining the PARPi rucaparib with KP372-1 in NSCLC, pancreatic cancer, and breast cancer cells, which were reconstituted or knocked out for NQO1 expression, treated with or without dicoumarol (**Figures 1F–K**). A549 NSCLC and MiaPaCa-2 pancreatic cancer cells expressing significant KRAS-driven NQO1 levels were hyper-sensitive to treatment with the PARPi rucaparib + KP372-1, but this sensitivity disappeared when the cancer cells were treated with the NQO1 inhibitor DIC (**Figures 1F, G**). Conversely, CRISPR/Cas9-based generation of stable *NQO1* knockout of A549 and MiaPaCa-2 cells were significantly resistant to the drug alone or combination treatment (**Figures 1H, I**). *NQO1* deficient triple-negative breast cancer (TNBC) MDA-MB-231 cells were also inherently resistant to KP372-1, with or without the PARPi rucaparib (**Figure 1J**). By contrast, MDA-MB-231 cells were rendered hypersensitive to rucaparib ± KP372-1 after NQO1 expression was restored but once again became resistant when treated with DIC (**Figure 1K**). Furthermore, a similar *NQO1*-dependent toxicity was noted in MCF-7 cells treated with non-toxic dose of rucaparib (0.4 µM) and various doses of KP372-1 (0.025 – 0.2 µM) (**Figures S1K, L**). The significant synergistic effect of PARPi + KP372-1 was also confirmed *via* colony formation assay in A549 cells (**Figure S1M**). In all of the above studies *NQO1* knockout or re-expression in A549, MiaPaCa-2, and MDA-MB-231 cells was confirmed by western blotting (insert, **Figures 1H, I, K**). Our previous work demonstrated that A549, MCF-7, and MiaPaCa-2 cells harbor different oncogenic driver or passenger mutations (21). This suggests that rucaparib + KP372-1 enhanced toxicity in *NQO1* positive cancers may be relatively independent of oncogenic drivers involved.

### Inhibition of PARP1 prevents KP372-1-induced PAR formation to reverse NAD^+^/ATP depletion

**Figure 2:**
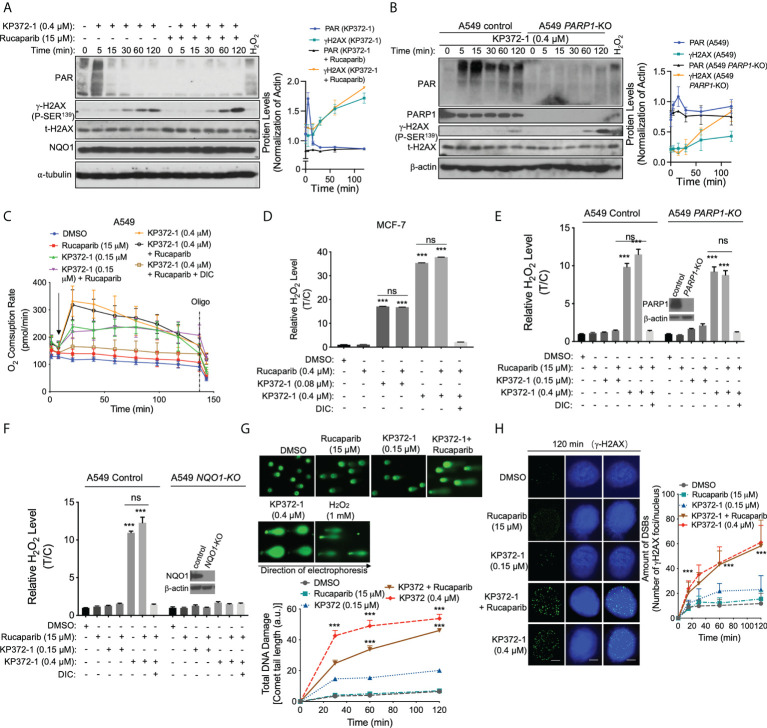
PARP Inhibition blocks KP372-1-induced PARP1 hyperactivation and amplifies DNA damage. **(A)** A549 cells were pre-treated ± rucaparib (15 µM, 2 h), then exposed to supra-lethal dose of KP372-1 (0.4 µM) ± rucaparib for indicated times, then PAR, γH2AX and NQO1 expression alterations were assessed and quantified. **(B)** A549 *PARP1* knockout cells were exposed to ± KP372-1 (0.4 µM) for 5 -120 min, western blot analysis of PAR and γH2AX formation at indicated time points. **(C)** A549 cells were pre-treated ± rucaparib (15 µM, 2 h), then exposed to KP372-1 (0.2 or 0.4 µM) ± rucaparib (added at t = 20 min, arrow), and real-time oxygen consumption rates (OCRs) were assessed by Seahorse XF analyses. Oligo, oligomycin. **(D)** MCF-7 cells were pre-treated ± rucaparib (0.4 μM, 2 h), then exposed to KP372-1 (0.08 or 0.4 µM) ± rucaparib for 2 h, relative H_2_O_2_ levels were assessed. **(E, F)** cells were pre-treated ± rucaparib (2 h), then exposed to rucaparib ± KP372-1 for 2 h, relative H_2_O_2_ levels were assessed in A549 *PARP1*-KO **(E)** and *NQO1*-KO **(F)** cells, **(G, H)** A549 cells were pre-treated ± rucaparib (2 h), then exposed to rucaparib ± KP372-1, cells were collected at indicated time points and assessed for: **(G)** Comet tail-lengths determined by alkaline comet assays; **(H)** DNA double strand breaks (DSBs) indicated by γH2AX with immunofluorescence staining. All error bars are means ± SD from three independent experiments. Scale bar indicates 10 µm. **(D–F)** ***P < 0.001 and ns: no significant, comparing each group or each data point with control (DMSO) treatments (*t* tests).

PARP proteins mediate post-translational PARylation of substrate proteins involved in processes such as transcription and DNA damage repair, among which PARP1 plays a particular important role in sensing DNA SSBs and DSBs (6). In A549 and MCF-7 cells, a rapid increase and continuous level of high-molecular-weight PARylated PARP1 protein (PAR-PARP1) was noted in 5 min after exposure to a lethal dose of KP372-1 (0.4 - 0.8 µM) alone, when hyperactivated PARP1 self‐parylates (**Figure S2A**). PAR-PARP1 (PAR) formation was a dynamic, time-dependent process that lasted approximately 15 minutes in which peak levels occurred at approximately 5 minutes, and was accompanied by the appearance of γH2AX protein, a marker of DNA DSBs (**Figure 2A** and **Figure S2B**). Co-addition of the PARPi rucaparib (15 µM or 0.4 µM) dramatically suppressed PAR formation (**Figure S2A**) and induced significantly greater amounts of γH2AX protein (**Figure 2A** and **Figure S2B**). Moreover, the synthetic treatment had no effect on the NQO1 protein expression levels (**Figure 2A**). To further confirm whether PARP1 plays an essential role in the combination therapy of PARPi + KP372-1, CRISPR/Cas9-based generation of stable *PARP1* knockout of A549 (**Figure S2C**) and shRNA *PARP1* knockdown of MCF-7 (**Figure S2D**) cells were examined for cell viabilities after exposure to various doses of KP372-1 with or without rucaparib (15 µM or 0.4 µM). Consistently, enhanced lethality was noted in A549 *PARP1*-KO (**Figure S2C**) and MCF-7 sh*PARP1* (**Figure S2D**) cells. The suppression of PAR formation was confirmed by western blot analysis and earlier and greater amounts of γH2AX protein was observed in A549 *PARP1*-KO cells (**Figure 2B**). Here we observed that insertion of vector caused a little bit insensitivity of A549 cells to KP372-1 resulting in sustained PARylation in control cells, which was different from wild type (**Figure 2B** and **Figure S2C**). All together, these data suggest that treatment with KP372-1 induces DNA DSBs, and PARP1 inhibition results in the increase of this DNA damage.

PARP1 catalyzes polymerization of ADP-ribose units from donor NAD^+^ molecules on target proteins, resulting in PAR formation (35, 36). To investigate whether PARPi alters KP372-1-induced ATP levels, we examined NAD^+^ and ATP levels in MCF-7 cells (**Figure S2E**) or A549 *PARP1*-KO cells (**Figures S2F, G)** after exposure to sublethal or lethal dose of KP372-1 with or without rucaparib. Interestingly, exposure of MCF-7 or A549 cells to a lethal dose of KP372-1 resulted in significant NAD^+^ and ATP losses, while PARP inhibition by rucaparib or *PARP1*-knockout rescued these losses (**Figures S2E–G**), consistent with suppression of PARP1 activity/hyperactivation monitored by PAR formation. Together, these results indicate that inhibition of PARP1 hyperactivation is required to KP372-1-mediated enhanced synergistic lethality.

### PARP inhibition amplifies *NQO1*-dependent DNA damage induced by KP372-1

Previous study has shown that *NQO1*-dependent futile redox cycling oxidizes NAD(P)H to create reactive oxygen species (ROS) very quickly (37). To determine whether KP372-1 ± PARPi affect ROS generation, we examined oxygen consumption rates (OCRs) and ROS generation by measurement of H_2_O_2_ level. As expected, exposure of A549 cells to a sublethal dose of KP372-1 alone (0.15 µM) or in combination with synergistic doses of rucaparib (15 µM), resulted in equivalent OCRs, suggesting that these doses of KP372-1 caused significant cell stress, but cells were able to keep up with the demand for NAD(P)H/NAD(P)^+^, without PARP1 hyperactivation. At the higher KP372-1 dose (0.4 µM) or combined with rucaparib, NQO1 futile redox becomes exhausted resulting in a decay of OCRs (**Figure 2C**). Consistently, the similar results were found in MiaPaCa-2 and MCF-7 cells (**Figures S3A, B**). Moreover, exposure of MCF-7 cells to KP372-1 (0.08 or 0.4 µM) ± rucaparib resulted in a significant increase in H_2_O_2_ levels compared to untreated group, and DIC suppressed these treatments induced H_2_O_2_ production (**Figure 2D** **).** Consistently, a similar result was obtained in A549 cells (**Figure S3C**). In addition, *PARP1* knockout had no significant effect on KP372-1 ± rucaparib induced H_2_O_2_ levels compared to A549 parental cells (**Figure 2E**), while NQO1 knockout totally blocked the H_2_O_2_ production (**Figure 2F**), indicating that KP372-1 ± rucaparib induced ROS generation is NQO1-dependent. ROS-induced cell stress has been suggested to induce DNA damage (38, 39). We next assessed the total DNA damage using alkaline comet assay which can detect base damage, SSBs, and DSBs. The combination treatment of KP372-1 (0.15 µM) with rucaparib (15 µM) resulted in a large and statistically significant enhancement of comet tail length to 40 ± 10 a.u. compared to controls or individual single treatments (**Figure 2G**). To determine the specific DNA damage induced by KP372-1 and the PARPi, we next analyzed Ser139-phosphorylated γH2AX foci, which are considered to be an early response to DNA DSBs (40). As expected, exposure of A549 cells to non-toxic dose of rucaparib or sublethal dose of KP372-1 alone had no significant effect on γH2AX foci compared with DMSO treatment, whereas combined treatment with these two agents resulted in a dramatic increase in γH2AX foci formation, which was equivalent to the over-lethal dose of KP372-1-induced foci formation (**Figure 2H** and **Figure S3D**). Together, these results suggest that KP372-1 induces cell stress *via* ROS generation resulting in an increase of DNA DSBs and promotes PARP inhibitor-induced cytotoxicity.

### Disturbance of intracellular calcium homeostasis by KP372-1 induces AKT hyperactivation

**Figure 3:**
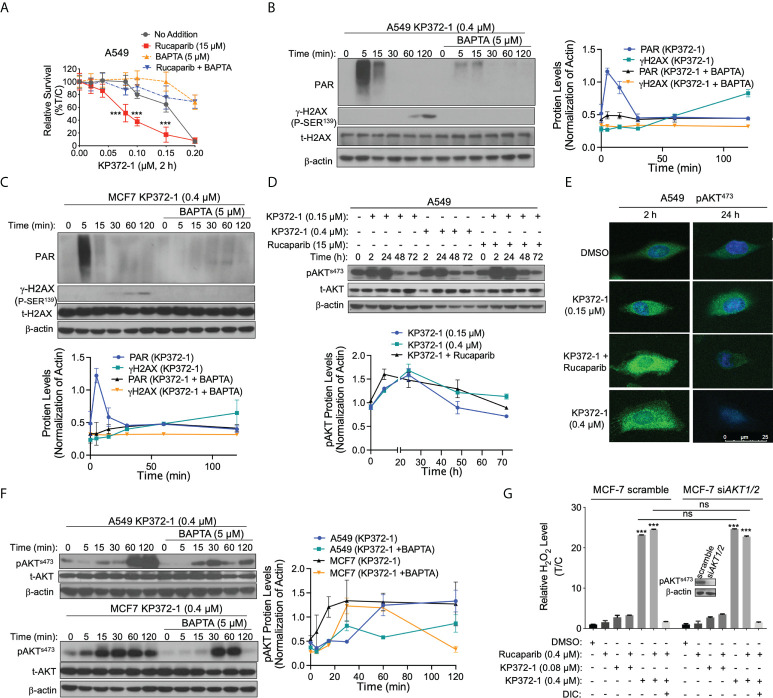
KP372-1 causes Ca^2+^ releasing and AKT hyperactivation to enhance the lethality of PARP inhibitor. **(A)** Relative survival assay in A549 NSCLC cells treated with BAPTA (5 μM) under conditions of KP372-1 ± rucaparib. Cells were pre-treated ± rucaparib (15 µM) for 1 h, then added ± BAPTA (5 µM, 1 h), and then exposed to KP372-1 ± rucaparib ± BAPTA for 2 h, followed by washing and replacing media, cell viability was assessed 7 days later. **(B, C)**, PAR and γH2AX alterations were assessed and quantified in A549 NSCLC **(B)** and MCF-7 cells **(C)**. **(D, E)** A549 cells were pre-treated ± rucaparib (15 µM, 2 h), then exposed to KP372-1 ± rucaparib for 2 h, followed by washing and replacing media, cells were collected at indicated time points and assessed for: **(D)** Levels of pAKT^s473^ and total AKT (t-AKT), and bottom panel showed quantification of pAKT^s473^; **(E)** Fluorescence image of pAKT^s473^. **(F)** Cells were treated as **(B, C)** then pAKT^s473^ levels alterations were assessed and quantified in A549 NSCLC and MCF-7 cells. **(G)** MCF-7 scramble and si*AKT1/2* cells were treated as **(D)** then cells were collected at 2 h and relative H_2_O_2_ in MCF-7 cells were determined. Results were separately repeated at least three times, *AKT* knockdown efficiency for **(E)** was confirmed by Western blot in **(G)**. All error bars are means ± SD. **(A)** *** *P* < 0.001, comparing each data point with KP372-1 alone treatments (grey color) (*t* tests). **(F)**, *** *P* < 0.001 and ns: no significant, comparing each group with control (DMSO) treatments (*t* tests).

Several studies have shown that ROS regulates calcium releasing from ER to cytoplasm (41, 42). To determine whether KP372-1 or combination treatment interrupts intracellular ion homeostasis, we investigated their impacts on intracellular calcium using BAPTA-AM, a chelator of intracellular calcium pool. As hypothesized, a non-lethal dose of BAPTA-AM pretreatment significantly spared KP372-1- or combination treatment-induced lethality in *NQO1^+^* A549 cells (**Figure 3A** and **Figures S4A, B**). Next, to further validate the effect of calcium induced by KP372-1 or combination treatment on cell growth, we examined PARP1 activity estimated by detection of PAR formation, and DNA DSB indicated *via* γH2AX, respectively. Consistently, PAR formation and γH2AX levels were significantly suppressed by BAPTA-AM in A549 and MCF-7 cells (**Figures 3B, C**). Together, these data indicate that a lethal dose of KP372-1 or non-lethal dose of KP372-1 + PARPi treatment interrupts intracellular calcium homeostasis resulting in loss of cell growth control and cancer cell death.

AKT activation commonly occurs in human cancers and promotes PARPi resistance to cancer therapies (8), however, several reports have demonstrated that transient activation of AKT or AKT hyperactivation promotes cancer cell death (28–30). KP372-1 was reported to be a potent AKT inhibitor in several studies (24, 25). We therefore examined the effect of KP372-1 or rucaparib alone or combination treatment on AKT expression. Surprisingly, a 2 h treatment of *NQO1^+^* A549 cells with KP372-1 ± PARPi resulted in a dramatic increase in active AKT phosphorylated on serine 473 within 24 h, and then gradually returned to normal level by 72 h compared to untreated cells, and the combination treatment of KP372-1 and PARPi induced AKT hyperactivation more effectively compared to KP372-1 treatment alone (**Figure 3D**). This AKT hyperactivation observed on western blots was confirmed by the analysis of confocal fluorescence microscopy (**Figure 3E**), although it returned to normal level more quickly in the confocal images compared to western blot analysis (**Figures 3D, E** and **Figure S4C**). Furthermore, *PARP1* knockout had no effect on KP372-1 ± rucaparib induced AKT hyperactivation compared to A549 parental cells (**Figure S4D**), while *NQO1* knockout significantly suppressed the AKT hyperactivation (**Figure S4E**). The non-lethal dose of PARPi rucaparib only induced a slight AKT activation (**Figure S4F**). In addition, treatment with BAPT-AM partially blocked the AKT hyperactivation in A549 or MCF-7 cells (**Figure 3F**), suggesting AKT is a downstream of calcium signaling. We also knocked down *AKT* by siRNA to investigate whether AKT expression affects KP372-1-induced ROS formation. As shown, silencing *AKT* did not affect ROS generation compared to scramble group under treatment with KP372-1 ± PARPi (**Figure 3G**). To verify whether KP372-1 inhibits AKT activation, A549 or MCF-7 cells were exposed to KP372-1 ± PARPi for 24 h. Consistent with other reports (24, 25), western blot analysis showed that AKT levels were efficiently repressed either in A549 or MCF-7 cells (**Figure S4G**). Together, our findings suggest that the transient hyperactivity of AKT is required for the synergistic lethality of KP372-1 with PARP inhibitors in cancer cells.

### KP372-1 treatment overcomes PARP inhibitor resistance *via* inhibiting FOXO3a/GADD45α pathway

**Figure 4:**
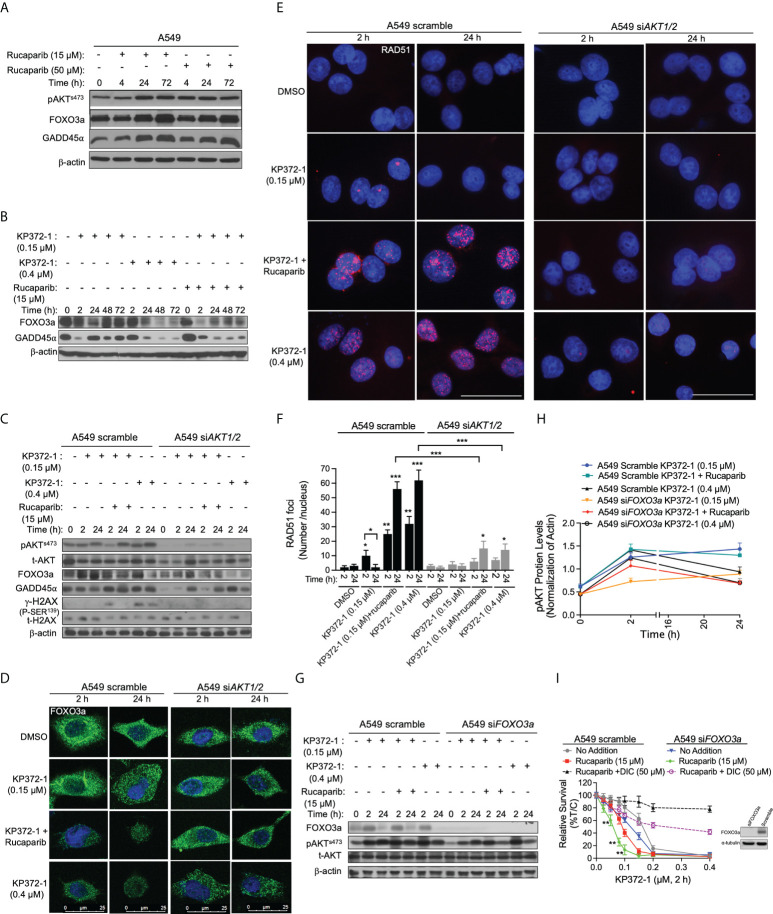
KP372-1 inhibits DNA repair to overcome resistance of PARP inhibitor. **(A)** A549 NSCLC cells were treated with rucaparib (15 µM or 50 µM) for 4 h, followed by washing and replacing fresh media, then cells were collected at indicated times and assessed for: pAKT^s473^, FOXO3a, and GADD45α alterations. **(B–G)** Cells were pre-treated ± rucaparib (15 μM, 2h), then exposed to KP372-1 ± rucaparib (15 μM) for 2 h followed by washing and replacing media, cells were collected at indicated timepoints and assessed for: **(B)** FOXO3a and GADD45α levels in A549 cells; **(C)** pAKT^s473^, t-AKT, FOXO3a, GADD45α, and γH2AX alterations in A549 scramble and si*AKT1/2* cells; **(D)** Fluorescence images of FOXO3a alterations in A549 scramble and si*AKT1/2* cells; **(E)** Quantification of RAD51 foci per nuclei in A549 scramble and si*AKT1/2* cells; **(F)**, Immunofluorescence staining of RAD51 expression in A549 scramble and si*AKT1/2* cells; **(G, H)** The expression and quantification of pAKT^s473^ in A549 scramble and si*FOXO3a* cells. **(I)** A549 scramble and si*FOXO3a* cells were pre-treated ± rucaparib (15 μM, 2h), then exposed to KP372-1 ± rucaparib (15 μM) for 2 h followed by washing and replacing media, cell viability was assessed after 7 days. The efficiency of *FOXO3a* knockdown was confirmed by Western blot analysis. All results were separately repeated at least three times. *AKT* knockdown efficiency for **(D–F)** was confirmed by Western blot shown in **(C)**. Scale bar indicates 25 µm **(D)** and 15 µm **(E)** respectively. Error bars are means ± SD. **(F)** * *P* < 0.05, **P < 0.01 and *** *P* < 0.001, comparing each group with control (DMSO) treatments (*t* tests). **(H)** **P < 0.01, comparing each data point with KP372-1+ rucaparib treatments in A549 scramble cells (red color) (*t* tests).

It has been suggested that PARPi activate AKT to induce resistance in cancer therapies (8), however, constitutive activation of AKT or AKT hyperactivation inhibits FOXO, leading to myeloid maturation and subsequent cell death in acute myeloid leukemia (AML) cells (29). FOXO3a, a member of the FOXO subfamily of forkhead transcription factors, plays a pivotal role in cellular stress responses and is implicated in DNA repair inhibition *via* downregulation of the growth arrest and DNA-damage-inducible protein 45 alpha (GADD45α) expression (43, 44). Therefore, we hypothesized that hyperactivation of AKT induced by KP372-1 could inhibit FOXO3a/GADD45α pathway and thereby potentially overcome PARPi resistance. To test that hypothesis, we first examined the effect of sublethal (15 µM) and lethal (50 µM) doses of rucaparib on the expression of AKT, FOXO3a and GADD45α over time. Western blot analysis showed that both doses of rucaparib increased the expression of these proteins after 24-72 h treatments, suggesting a potential recovery of DNA repair (**Figure 4A** and **Figures S5A–C**). Next, we examined the impact of KP372-1 or KP372-1 ± PARPi on the expression of FOXO3a and GADD45α over time. As hypothesized, the combination treatment of KP372-1 (0.15 µM) with rucaparib (15 µM) or lethal dose of KP372-1 (0.4 µM) alone significantly inhibited the expression of FOXO3a and GADD45α over time, while a sublethal does of KP372-1 (0.15 µM) alone caused minimal changes in the expression of these two proteins (**Figure 4B** and **Figures S5D–F**). We then knocked down *AKT* by siRNA to investigate whether AKT expression affects KP372-1 ± PARPi treatment-induced FOXO3a/GADD45α inhibition. The analysis of western blot and confocal fluorescence microscopy data showed that siRNA-mediated knockdown of AKT efficiently recovered FOXO3a/GADD45α expression to basal levels of untreated cells (**Figures 4C, D** and **Figures S5G, H**), and completely abolished KP372-1 ± rucaparib induced γH2AX levels and reduced RAD51 expression, indicating a reduction in DNA DSBs (**Figures 4C, E, F**). Finally, knockdown of FOXO3a expression by siRNA had no effect on KP372-1 ± rucaparib-induced AKT hyperactivation (**Figures 4G, H**). To further confirm the role of FOXO3a/GADD45α in KP372-1 ± rucaparib induced cell death, we knocked down *FOXO3a* and *GADD45α* to assess cell viability. As expected, silencing FOXO3a or GADD45α significantly increased KP372-1+ PARPi-induced cell death (**Figure 4I** and **Figure S5I**). Together, all the above data suggest that KP372-1-induced transient AKT hyperactivation inhibits the downstream pathway of FOXO3a/GADD45α that blocks DNA repair and thereby overcomes PARP inhibitor resistance.

### Combination treatment of KP372-1 with PARP inhibitor induces cancer cell autophagy and apoptosis

**Figure 5:**
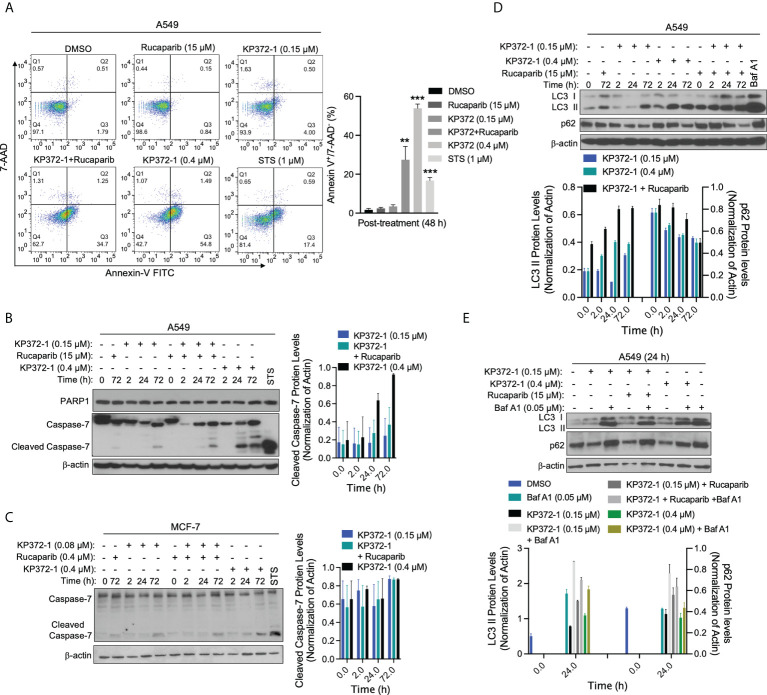
Combination of KP372-1 with PARP inhibitor induces cell autophagy and cell apoptosis. Cells were pre-treated ± rucaparib (0.4 µM or 15 µM, 2 h), then exposed to KP372-1 ± rucaparib or KP372-1 ± rucaparib ± Bafilomycin A1 (Baf A1) for 2 h followed by washing and replacing media; cells treated with Baf A1 (0.05 µM) were kept with Baf A1 for 24 h; positive control cells were exposed to Staurosporine (STS, 1 μM) for 18 h; then cells including debris in media were finally collected at indicated timepoints and examined for: **(A)** Annexin-V/7-AAD staining to determine cell death way *via* flow cytometry, early apoptosis part indicated by Annexin-V^+^/7-AAD^-^ was quantified on the left pannel; **(B)** PARP1 and cleaved caspase 7 alterations in A549 cells; **(C)** Levels of cleaved caspase 7 in MCF-7 cells; **(D, E)** LC3 I/II and p62 levels in A549 cells. Results were separately repeated at least three times and protein levels were quantified. **(A)** Error bars are means ± SD. ** *P* < 0.01 *** *P* < 0.001, comparing each group with control (DMSO) treatment (*t* tests).

Since non-lethal dose of combination treatment of KP372-1 with PARPi resulted in enhanced lethality (**Figures 1E–K** and **Figure S1L**), we investigated the cell death pathways being activated by this lethal combination treatment. Several studies suggest that KP372-1 induces apoptosis in acute myelogenous leukemia and head and neck cancer cells (24, 25). In our study, flow cytometry analysis revealed that a non-lethal dose of KP372-1 (0.15 µM) or rucaparib (15 µM) alone had no apparent effect on A549 cancer cell growth, while this combination treatment or a lethal dose of KP372-1 (0.4 µM) caused significant apoptosis induction (Annexin-V^+^/7ADD^-^) by 48 h (**Figure 5A**). In addition, typical caspase-mediated cleavage of caspase-7 proteolysis was observed under the conditions of a non-lethal dose of KP372-1 with rucaparib or a lethal dose of KP372-1 treatment by 24 h in A549 and MCF-7 cells (**Figures 5B, C**). However, data from several investigators indicate that there are multiple cell death pathways activated in cancers and even multiple pathways occurring simultaneously in the same cell (45, 46). We have clear evidence of apoptosis being induced in our system but another pathway that can be induced by oxidative stress is autophagic cell death (47). To investigate whether our treatments induce autophagy, we examined the protein levels of microtubule-associated protein 1A/1B-light chain 3 (LC3), especially LC3-phosphatidylethanolamine conjugate (LC3 II), a widely used marker to monitor autophagy and autophagy-related processes (48). We found that low dose of KP372-1 (0.15 µM) induced the elevated LC3 II after 72 h treatment in A549 cells, while rucaparib was added with 0.15 µM KP372-1, LC3 II up-regulation was noted after 2 h treatment and sustained for 72 h (**Figure 5D**). Combined exposure of these two agents was not statistically different from exposure to a lethal dose of KP372-1 (0.4 µM) (**Figure 5D**). Since LC3 II accumulation could be due to either autophagy induction or inhibition of autophagic flux, we further examined the change of p62 protein. The p62 protein, a classical receptor of autophagy, is itself degraded by autophagy in lysosome and accumulates when autophagy is inhibited (49). We found that exposure of A549 cells to either KP372-1 (0.15 or 0.4 µM) or combination treatment induced a reduction of p62 levels (**Figures 5D, E**), while co-addition of KP372-1 ± rucaparib with Bafilomycin A1 (Baf A1), which used as an inhibitor of autophagosome-lysosome fusion to determine the activity of autophagic flux (50), caused both p62 and LC3 II accumulation (**Figure 5E**), indicating an autophagy induction by KP372-1 ± rucaparib treatment. Together, these data suggest that KP372-1 ± PARPi treatment appears to induce autophagy and then either switches to or is accompanied by induction of apoptosis and cell death.

### Synergistic treatment enhances accumulation of both agents in tumors resulting in super-additive antitumor activity in orthotopic pancreatic and NSCLC models

**Figure 6:**
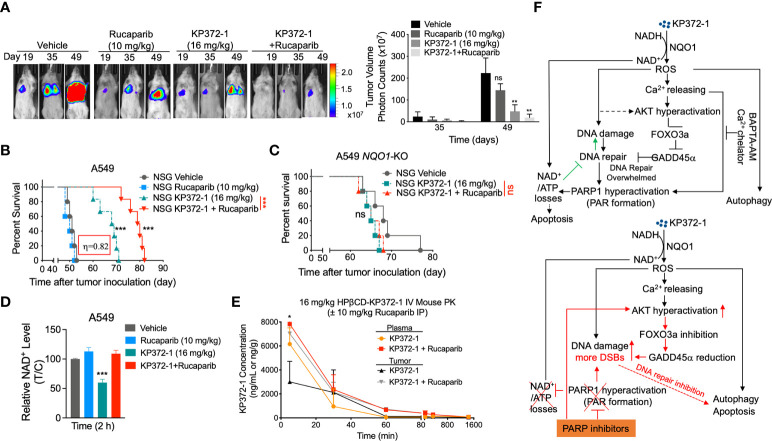
KP372-1 synergizes with PARP inhibitor against orthotopic A549 and *NQO1*-KO NSCLC xenografts. **(A–C)** Orthotopic A549 and *NQO1* knockout (A549 *NQO1*-KO) tumors were established in 20-22 g female NSG mice by injecting 1 x 10^6^ (A549) or 1.1 x 10^6^ (A549 *NQO1*-KO) cells/mouse into lung *via* intravenous tail vein. After two weeks, mice were treated with/without rucaparib (10 mg/kg, i.p.) for 2 h followed by HPβCD (Vehicle) or HPβCD-KP372-1 (KP372-1) (16 mg/kg, i.v.) every other day for 5 injections. Experiments were repeated at least two times, *n* = 5/group. Images of representative mouse tumors at indicated times and quantified tumor volumes (right panel). **(B)** Kaplan-Meier survival curves of A549 orthotopic mice. **(C)** Kaplan-Meier survival curves of A549 *NQO1*-KO orthotopic mice. **(D)** Orthotopic A549 tumor-bearing female NSG mice (*n* = 3/group) were treated as in **(A)** and sacrificed at 2 h, relative NAD^+^ levels of tumor tissues were determined. **(E)** Pharmacokinetics (PK) of KP372-1 in orthotopic A549 tumor-bearing female NSG mice (*n* = 3/group) treated as in **(A)** and sacrificed at indicated times. **(F)** summary description of this study. Data are shown as mean ± SD. *P < 0.05, **P < 0.01, and ***P < 0.001, ns: no significant, comparing each data point with those of vehicle treatments determined by unpaired Student’s *t*-test **(A, D, E)** or log-rank test in **(B, C)**. **(B)** Synergy values (η = 0.82) were reported based on multiple dose responses, or on comparative *p* values indicated.

To test the *in vivo* efficacy of the KP372-1 ± PARPi treatment, we established orthotopic NSCLC tumor xenografts by injecting ~1x10^6^ A549-Luciferase cells per mouse by tail vein into NSG mice. After 7 days, mice were randomly divided into different groups (*n* = 5/group) and treated with vehicle (HPβCD, intravenously [i.v.], tail vein), HPβCD-KP372-1 (16 mg/kg, i.v.) or rucaparib (10 mg/kg, Intraperitoneal [i.p.]) alone, or rucaparib (10 mg/kg, i.p.) 2 h prior to KP372-1 (16 mg/kg, i.v.). Treatments were given every other day, for a total of 5 treatments. Mice were then monitored for changes in tumor volumes (**Figure 6A**), weight loss (**Figure S6A**), overall survival (**Figures 6B, C**), and NAD^+^ loss (**Figure 6D**). Bioluminescence imaging (BLI) and overall survival showed that non-toxic dose of rucaparib had no significant effects on tumor-growth suppression, while KP372-1 alone resulted in decreased tumor growth and enhanced the survival rate of human A549 tumor-bearing mice, although all mice succumbed to tumor burden by day 72 (**Figures 6A, B**). In contrast, mice treated with KP372-1 + rucaparib showed enhanced antitumor activity and significant survival benefit compared to these two single agents alone (**Figures 6A, B**). To test for *NQO1* dependence on this enhanced *in vivo* toxicity, we established xenografts with A549 *NOQ1*-KO cells in NSG mice and treated them with either KP372-1 treatment alone or with KP372-1 + PARPi combination treatment and observed no enhancement of KP372-1 + rucaparib in tumor killing and no increase in survival (**Figure 6C**), suggesting that KP372-1 alone or KP372-1 + rucaparib mediated antitumor efficacy is dependent on *NQO1 in vivo*. In addition, we found that treatment with KP372-1 alone caused a dramatic decrease in NAD^+^ levels, but treatment with KP372-1 + rucaparib did not decrease NAD^+^, which is consistent with our observations *in vitro* (**Figure 6D**).

To investigate whether similar *in vivo* survival benefits could be achieved in another human cancer model, we established pancreatic orthotopic tumors in NSG mice by directly injecting ~1x10^6^ MiaPaCa-2-Luciferase cells into pancreas. Similar improved antitumor efficacies were noted in KP372-1 alone or rucaparib + KP372-1-treated mice, and the combination treatment dramatically extended mouse life span compared to KP372-1 treatment alone (**Figures S6B, C**). No significant mice weight losses were observed in these two models (**Figures S6A, D**), indicating the doses of these agents had no overt toxic effects on mice.

To investigate whether KP372-1 combined with rucaparib affects the individual pharmacokinetic (PK) profiles of the two drugs, we tested the drug PKs in the tumor, plasma, liver, and brain of A549 NSCLC orthotopic tumor bearing mice. The treatment doses and schedules were defined according to the maximum tolerated doses (MTD) determined in the model used for this study. PK analyses revealed no alterations of KP372-1 concentrations in blood (plasma), brain, or liver of mice treated with KP372-1 combined with rucaparib compared to KP372-1 treatment alone (**Figure 6E** and **Figure S6E**). However, the combination treatment significantly increased the KP372-1 concentrations in tumor tissues compared to single-agent treatment (**Figure 6E**), and rucaparib levels were significantly elevated in tumor compared to plasma or brain tissue over time (**Figure S6F**), which has also been reported by Murray et al. (51). These data suggest that KP372-1 or PARP inhibitor rucaparib alone moves to target tissues with limited efficiency, while combination treatment efficiently enhances accumulation of both drugs to tumor tissues.

## Discussion

Recent studies suggested KP372-1 as a promising and potent *NQO1*-dependent anti-tumor agent that induced dramatic ROS generation and DNA damage, leading to PARP1 hyperactivation and a decrease in NAD^+^/NADH redox state, which suppressed tumor cell growth *in vitro* and *in vivo* (26, 27). In this study, we show that combining PARPi with KP372-1 leads to synergistic antitumor effect with non-toxic doses of both drugs in *NQO1* overexpressing cancer cells. KP372-1 + PARPi treatment results in robust, *NQO1*-dependent, tumor-selective induction of DNA DSBs, autophagy and apoptosis both *in vitro* and *in vivo*. Mechanistically, as shown in **Figure 6F**, KP372-1 is reduced by NQO1 in the presence of NAD(P)H, resulting in superoxide (ROS formation), which then induces calcium releasing into cytoplasm and DNA damage. The increased calcium concentration in cytoplasm and ROS production promotes AKT hyperactivation that blocks DNA repair *via* inhibition of the FOXO3a/GADD45a pathway. This results in the accumulation of DNA damage and PARP1 hyperactivation which leads to NAD^+^/ATP loss and the cells undergo a caspase-mediated apoptosis and autophagy. However, when KP372-1 combines with PARPi (rucaparib), the PARPi blocks the KP372-1-induced PARP1 hyperactivation and rescues NAD^+^/ATP depletion, resulting in more DNA damage and higher AKT hyperactivation. This AKT hyperactivation further inhibits FOXO3a/GADD45α pathway resulting in even greater DNA DSBs induction, and cells undergo tumor-selective and *NQO1*-dependent autophagy and apoptosis.

Here, we show that KP372-1 combined with PARPi resulted in enhanced toxicity and synergistic killing of *NQO1^+^* cancers through a robust ROS induction and enhanced DNA damage response. Interestingly, cells exposed to KP372-1 exhibited dramatic and transient activation of AKT (AKT hyperactivation) instead of AKT inhibition. AKT hyperactivation is suggested to sensitize cells to ROS-induced apoptosis (28, 30). Growing evidence implies that multiple cell death pathways can occur in parallel in cancer cells (45, 46). In our study, we found that exposure of *NQO1^+^* cancer cells to KP372-1 alone caused PARP1 hyperactivation and NAD^+^/ATP depletion, and cells underwent autophagy and capase-7 dependent apoptosis, while exposure of these cells to KP372-1 + PARP inhibitor rucaparib induced elevated ROS formation and inhibition of DNA repair resulting in rapidly autophagic and apoptotic cell death (**Figures 2G, H**, **4E**, **5B–E**, and **Figures S2E–G**). Furthermore, we show that KP372-1 was ten-fold more potent to kill *NQO1*-positive cancer cells (IC50: 0.017 µM vs. 2 µM) compared to other NQO1 bioactivatable drugs, such as β-lapachone (**Figures 1E, F** and **Figures S1G, M**) (21). Similar to β-lapachone, KP372-1 exhibited little dependence on specific oncogenic driver or passenger mutations (21). MCF-7, MDA-MB-231- *NQO1^+^*, and MiaPaCa-2 cells, which have wild type vs mutant p53 and KRAS, respectively, were equally sensitized to KP372-1. This relative lack of dependence on specific oncogenic drivers has the potential to expand the efficacious use of KP372-1 in *NQO1^+^* solid cancers.

Our data clearly demonstrate that KP372-1 + PARPi-enhanced tumor-selective toxicity occurs in *NQO1^+^* cancer cells. Wild type MDA-MB-231 and A549 *NQO1*-KO cells, which have no or undetectable NQO1 expression, were resistant to KP372-1 + PARPi treatment. The synergy of KP372-1 + PARPi that increased efficacy in killing *NQO1^+^* cancer cells and enhanced KP372-1 effects on cancer cell death occurred through AKT hyperactivation and induction of increased DNA damage, and the combination therapy caused more autophagic cell death than KP372-1 treatment alone (**Figure 5D**). Interestingly, the enhanced toxicity and synergy observed with the non-lethal dose of KP372-1 and PARPi took time to develop were not immediately evident compared to lethal doses of KP372-1.

Our data indicate that the combination treatment of KP372-1 with PARPi has strong translational potential. While ongoing clinical trials of PARPi treatment have demonstrated promise in HR-deficient breast and ovarian tumors and recently been expanded to other solid tumors (52–54), the high rate of PARPi resistance has dampened the initial enthusiasm. Therefore, identifying potential drugs to overcome PARPi resistance is imperative. Several studies have shown that PARPi resistance involves enhanced DNA repair and cell cycle progression (3, 4). In this study, we reveal that PARPi resistance may involve recovery of DNA repair *via* AKT/FOXO3a/GADD45a pathway, and that treatment with KP372-1 blocked this pathway and efficiently overcame PARPi resistance. Based on our preclinical studies *in vivo*, the synergistic antitumor activity we observed *in vitro* was confirmed in NSCLC and pancreatic cancer mouse tumor models which significantly improved overall survival of the mice treated with the KP372-1 + PARPi combination with no increase in toxicity to normal tissues. High dose of KP372-1 (25 mg/kg) treatment caused extremely low side effect of methemoglobinemia (i.e., labored breathing, lethargy in 45 min) compared to β-lapachone, and KP372-1 + PARPi showed no signs of methemoglobinemia. Moreover, pharmacokinetics analysis reveals that the KP372-1 + PARPi combination therapy enhanced the accumulation of both agents in tumors (**Figure 6E** and **Figures S6E, F**), while β-lapachone + PARPi only increased the PARPi but not β-lapachone concentration in tumors (21), suggesting that KP372-1 may be ideally suited to exploit synergy with DNA repair inhibitors. Our study indicates that treatment with KP372-1 is an effective, potential therapeutic strategy to expand PARP inhibitor clinical utility and to overcome any developing PARPi resistance.

## Data availability statement

Publicly available datasets were analyzed in this study. This data can be found here: TCGA database.

## Ethics statement

The animal study was reviewed and approved by the Indiana University IACUC committee.

## Author contributions

LJ and XH designed the experiments, analyzed the data, and wrote the first draft manuscript. LJ, XS, YZ and ST performed experiments. LJ, YL and JW performed the animal experiments. XW synthesized KP372-1. JK and NW performed the PK analysis. LJ, YC, MM, TL, Y-XF and XH reviewed and edited the manuscript. XH supervised the project. All authors contributed to the article and approved the submitted version.

## Funding

This work was supported by NIH/NCI grants (R01 CA221158-04, R01 CA224493-03, and R01 CA240952-01 to XH).

## Acknowledgments

We thank Dr. Hal Broxmeyer’s lab for their help with the analysis of OCR. We thank the IU Simon Comprehensive Cancer Center for the use of the Tissue Procurement & Distribution Core, which provided breast and lung cancer patient samples. We thank the IU School of Medicine animal breeding core facility. We thank IU Simon Cancer CCSG grant P30 CA087209, corresponding cores of Flow cytometry and biostatistics, and the Bone and Body Composition Core of the Indiana Clinical Translational Sciences Institute (CTSI).

## Conflict of interest

The authors declare that the research was conducted in the absence of any commercial or financial relationships that could be construed as a potential conflict of interest.

## Publisher’s note

All claims expressed in this article are solely those of the authors and do not necessarily represent those of their affiliated organizations, or those of the publisher, the editors and the reviewers. Any product that may be evaluated in this article, or claim that may be made by its manufacturer, is not guaranteed or endorsed by the publisher.

## References

[1] Mirza MR Pignata S Ledermann JA . Latest clinical evidence and further development of PARP inhibitors in ovarian cancer. Ann Oncol (2018) 29(6):1366–76. doi: 10.1093/annonc/mdy174 29750420

[2] Gourley C Balmana J Ledermann JA Serra V Dent R Loibl S . Moving from poly (ADP-ribose) polymerase inhibition to targeting DNA repair and DNA damage response in cancer therapy. J Clin Oncol (2019) 37(25):2257–69. doi: 10.1200/JCO.18.02050 31050911

[3] Noordermeer SM van Attikum H . PARP inhibitor resistance: A tug-of-War in BRCA-mutated cells. Trends Cell Biol (2019) 29(10):820–34. doi: 10.1016/j.tcb.2019.07.008 31421928

[4] Li H Liu ZY Wu N Chen YC Cheng Q Wang J . PARP inhibitor resistance: the underlying mechanisms and clinical implications. Mol Cancer (2020) 19(1):107. doi: 10.1186/s12943-020-01227-0 32563252 PMC7305609

[5] Herceg Z Wang ZQ . Functions of poly(ADP-ribose) polymerase (PARP) in DNA repair, genomic integrity and cell death. Mutat Res (2001) 477(1-2):97–110. doi: 10.1016/S0027-5107(01)00111-7 11376691

[6] Ray Chaudhuri A Nussenzweig A . The multifaceted roles of PARP1 in DNA repair and chromatin remodelling. Nat Rev Mol Cell Biol (2017) 18(10):610–21. doi: 10.1038/nrm.2017.53 PMC6591728 28676700

[7] Barkauskaite E Jankevicius G Ahel I . Structures and mechanisms of enzymes employed in the synthesis and degradation of PARP-dependent protein ADP-ribosylation. Mol Cell (2015) 58(6):935–46. doi: 10.1016/j.molcel.2015.05.007 26091342

[8] Gallyas F Jr. Sumegi B Szabo C . Role of akt activation in PARP inhibitor resistance in cancer. Cancers (Basel) (2020) 12(3):532. doi: 10.3390/cancers12030532 PMC7139751 32106627

[9] Tapodi A Bognar Z Szabo C Gallyas F Sumegi B Hocsak E . PARP inhibition induces akt-mediated cytoprotective effects through the formation of a mitochondria-targeted phospho-ATM-NEMO-Akt-mTOR signalosome. Biochem Pharmacol (2019) 162:98–108. doi: 10.1016/j.bcp.2018.10.005 30296409

[10] Yudushkin I . Getting the akt together: Guiding intracellular akt activity by PI3K. Biomolecules (2019) 9(2):1–14. doi: 10.3390/biom9020067 PMC6406913 30781447

[11] Stemke-Hale K Gonzalez-Angulo AM Lluch A Neve RM Kuo WL Davies M . An integrative genomic and proteomic analysis of PIK3CA, PTEN, and AKT mutations in breast cancer. Cancer Res (2008) 68(15):6084–91. doi: 10.1158/0008-5472.CAN-07-6854 PMC2680495 18676830

[12] Millis SZ Ikeda S Reddy S Gatalica Z Kurzrock R . Landscape of phosphatidylinositol-3-Kinase pathway alterations across 19784 diverse solid tumors. JAMA Oncol (2016) 2(12):1565–73. doi: 10.1001/jamaoncol.2016.0891 27388585

[13] Brett E Johnson TM Hong C Barnes M Aihara K McLean CY . Mutational analysis reveals the origin and therapy-driven evolution of recurrent glioma. Science (2014) 343(6167):189–93. doi: 10.1126/science.1239947 PMC3998672 24336570

[14] Stegeman H Span PN Kaanders JH Bussink J . Improving chemoradiation efficacy by PI3-K/AKT inhibition. Cancer Treat Rev (2014) 40(10):1182–91. doi: 10.1016/j.ctrv.2014.09.005 25312653

[15] Szanto A Hellebrand EE Bognar Z Tucsek Z Szabo A Gallyas F Jr. . PARP-1 inhibition-induced activation of PI-3-kinase-Akt pathway promotes resistance to taxol. Biochem Pharmacol (2009) 77(8):1348–57. doi: 10.1016/j.bcp.2009.01.008 19426673

[16] Ethier C Tardif M Arul L Poirier GG . PARP-1 modulation of mTOR signaling in response to a DNA alkylating agent. PloS One (2012) 7(10):e47978. doi: 10.1371/journal.pone.0047978 23110147 PMC3480502

[17] Liu Q Turner KM Alfred Yung WK Chen K Zhang W . Role of AKT signaling in DNA repair and clinical response to cancer therapy. Neuro Oncol (2014) 16(10):1313–23. doi: 10.1093/neuonc/nou058 PMC4165418 24811392

[18] Ibrahim YH Garcia-Garcia C Serra V He L Torres-Lockhart K Prat A . PI3K inhibition impairs BRCA1/2 expression and sensitizes BRCA-proficient triple-negative breast cancer to PARP inhibition. Cancer Discovery (2012) 2(11):1036–47. doi: 10.1158/2159-8290.CD-11-0348 PMC5125254 22915752

[19] Yap TA Kristeleit R Michalarea V Pettitt SJ Lim JSJ Carreira S . Phase I trial of the PARP inhibitor olaparib and AKT inhibitor capivasertib in patients with BRCA1/2- and non-BRCA1/2-Mutant cancers. Cancer Discovery (2020) 10(10):1528–43. doi: 10.1158/2159-8290.CD-20-0163 PMC7611385 32532747

[20] Ross D Kepa JK Winski SL Beall HD Anwar A Siegel. D . NAD(P)H:quinone oxidoreductase 1 (NQO1): chemoprotection, bioactivation, gene regulation and genetic polymorphisms. Chem Biol Interact (2000) 129(1-2):77–97. doi: 10.1016/S0009-2797(00)00199-X 11154736

[21] Huang X Motea EA Moore ZR Yao J Dong Y Chakrabarti G . Leveraging an NQO1 bioactivatable drug for tumor-selective use of Poly(ADP-ribose) polymerase inhibitors. Cancer Cell (2016) 30(6):940–52. doi: 10.1016/j.ccell.2016.11.006 PMC5161231 27960087

[22] Li LS Reddy S Lin ZH Liu S Park H Chun SG . NQO1-mediated tumor-selective lethality and radiosensitization for head and neck cancer. Mol Cancer Ther (2016) 15(7):1757–67. doi: 10.1158/1535-7163.MCT-15-0765 PMC5123441 27196777

[23] Zhang K Chen D Ma K Wu X Hao H Jiang S . NAD(P)H:Quinone oxidoreductase 1 (NQO1) as a therapeutic and diagnostic target in cancer. J Med Chem (2018) 61(16):6983–7003. doi: 10.1021/acs.jmedchem.8b00124 29712428

[24] Mandal M Younes M Swan EA Jasser SA Doan D Yigitbasi O . The akt inhibitor KP372-1 inhibits proliferation and induces apoptosis and anoikis in squamous cell carcinoma of the head and neck. Oral Oncol (2006) 42(4):430–9. doi: 10.1016/j.oraloncology.2005.09.011 PMC1414640 16442835

[25] Zeng Z Samudio IJ Zhang W Estrov Z Pelicano H Harris D . Simultaneous inhibition of PDK1/AKT and fms-like tyrosine kinase 3 signaling by a small-molecule KP372-1 induces mitochondrial dysfunction and apoptosis in acute myelogenous leukemia. Cancer Res (2006) 66(7):3737–46. doi: 10.1158/0008-5472.CAN-05-1278 16585200

[26] Zhao Y Hu Q Cheng F Su N Wang A Zou Y . SoNar, a highly responsive NAD+/NADH sensor, allows high-throughput metabolic screening of anti-tumor agents. Cell Metab (2015) 21(5):777–89. doi: 10.1016/j.cmet.2015.04.009 PMC4427571 25955212

[27] Viera T Patidar PL . DNA Damage induced by KP372-1 hyperactivates PARP1 and enhances lethality of pancreatic cancer cells with PARP inhibition. Sci Rep (2020) 10(1):20210. doi: 10.1038/s41598-020-76850-4 33214574 PMC7677541

[28] Nogueira V Park Y Chen CC Xu PZ Chen ML Tonic I . Akt determines replicative senescence and oxidative or oncogenic premature senescence and sensitizes cells to oxidative apoptosis. Cancer Cell (2008) 14(6):458–70. doi: 10.1016/j.ccr.2008.11.003 PMC3038665 19061837

[29] Sykes SM Lane SW Bullinger L Kalaitzidis D Yusuf R Saez B . AKT/FOXO signaling enforces reversible differentiation blockade in myeloid leukemias. Cell (2011) 146(5):697–708. doi: 10.1016/j.cell.2011.07.032 21884932 PMC3826540

[30] Nogueira V Patra KC Hay N . Selective eradication of cancer displaying hyperactive akt by exploiting the metabolic consequences of akt activation. Elife (2018) 7:1–23. doi: 10.7554/eLife.32213 PMC5980228 29687779

[31] Huang X Dong Y Bey EA Kilgore JA Bair JS Li LS . An NQO1 substrate with potent antitumor activity that selectively kills by PARP1-induced programmed necrosis. Cancer Res (2012) 72(12):3038–47. doi: 10.1158/0008-5472.CAN-11-3135 PMC4795165 22532167

[32] Chou T-C Talalay P . Quantitative analysis of dose-effect relationships: the combined effects of multiple drugs or enzyme inhibitors. Adv Enzyme Regulation (1984) 22:27–55. doi: 10.1016/0065-2571(84)90007-4 6382953

[33] Lee JJ Kong M Ayers GD Lotan R . Interaction index and different methods for determining drug interaction in combination therapy. J Biopharm Stat (2007) 17(3):461–80. doi: 10.1080/10543400701199593 17479394

[34] Gonzalez-Billalabeitia E Seitzer N Song SJ Song MS Patnaik A Liu XS . Vulnerabilities of PTEN-TP53-deficient prostate cancers to compound PARP-PI3K inhibition. Cancer Discovery (2014) 4(8):896–904. doi: 10.1158/2159-8290.CD-13-0230 24866151 PMC4125493

[35] Kim MY Zhang T Kraus WL . Poly(ADP-ribosyl)ation by PARP-1: ‘PAR-laying’ NAD+ into a nuclear signal. Genes Dev (2005) 19(17):1951–67. doi: 10.1101/gad.1331805 16140981

[36] Alemasova EE Lavrik OI . Poly(ADP-ribosyl)ation by PARP1: reaction mechanism and regulatory proteins. Nucleic Acids Res (2019) 47(8):3811–27. doi: 10.1093/nar/gkz120 PMC6486540 30799503

[37] Bey EA Reinicke KE Srougi MC Varnes M Anderson VE Pink JJ . Catalase abrogates beta-lapachone-induced PARP1 hyperactivation-directed programmed necrosis in NQO1-positive breast cancers. Mol Cancer Ther (2013) 12(10):2110–20. doi: 10.1158/1535-7163.MCT-12-0962 PMC3807805 23883585

[38] Cadet J Wagner JR . DNA Base damage by reactive oxygen species, oxidizing agents, and UV radiation. Cold Spring Harb Perspect Biol (2013) 5(2):1–18. doi: 10.1101/cshperspect.a012559 PMC3552502 23378590

[39] Srinivas US Tan BWQ Vellayappan BA Jeyasekharan AD . ROS and the DNA damage response in cancer. Redox Biol (2019) 25:101084. doi: 10.1016/j.redox.2018.101084 30612957 PMC6859528

[40] Podhorecka M Skladanowski A Bozko P . H2AX phosphorylation: Its role in DNA damage response and cancer therapy. J Nucleic Acids (2010) 2010:1–9. doi: 10.4061/2010/920161 PMC2929501 20811597

[41] Hool LC . Evidence for the regulation of l-type Ca2+ channels in the heart by reactive oxygen species: mechanism for mediating pathology. Clin Exp Pharmacol Physiol (2008) 35(2):229–34. doi: 10.1111/j.1440-1681.2007.04727.x 18197892

[42] Gorlach A Bertram K Hudecova S Krizanova O . Calcium and ROS: A mutual interplay. Redox Biol (2015) 6:260–71. doi: 10.1016/j.redox.2015.08.010 PMC4556774 26296072

[43] Liu Y Ao X Ding W Ponnusamy M Wu W Hao X . Critical role of FOXO3a in carcinogenesis. Mol Cancer (2018) 17(1):104. doi: 10.1186/s12943-018-0856-3 30045773 PMC6060507

[44] Tran H Brunet A Grenier JM Datta SR Fornace AJ Jr. DiStefano PS . DNA Repair pathway stimulated by the forkhead transcription factor FOXO3a through the Gadd45 protein. Science (2002) 296(5567):530–4. doi: 10.1126/science.1068712 11964479

[45] Fiers W Beyaert R Declercq W Vandenabeele P . More than one way to die: apoptosis, necrosis and reactive oxygen damage. Oncogene (1999) 18(54):7719–30. doi: 10.1038/sj.onc.1203249 10618712

[46] Bedoui S Herold MJ Strasser A . Emerging connectivity of programmed cell death pathways and its physiological implications. Nat Rev Mol Cell Biol (2020) 21(11):678–95. doi: 10.1038/s41580-020-0270-8 32873928

[47] Chen Y McMillan-Ward E Kong J Israels SJ Gibson SB . Oxidative stress induces autophagic cell death independent of apoptosis in transformed and cancer cells. Cell Death Differ (2008) 15(1):171–82. doi: 10.1038/sj.cdd.4402233 17917680

[48] Tanida I UT Kominami E . LC3 and autophagy. Methods Mol Biol (2008) 445:77–88. doi: 10.1007/978-1-59745-157-4_4 18425443

[49] Liu WJ Ye L Huang WF Guo LJ Xu ZG Wu HL . p62 links the autophagy pathway and the ubiqutin-proteasome system upon ubiquitinated protein degradation. Cell Mol Biol Lett (2016) 21:29. doi: 10.1186/s11658-016-0031-z 28536631 PMC5415757

[50] Mauthe M Orhon I Rocchi C Zhou X Luhr M Hijlkema KJ . Chloroquine inhibits autophagic flux by decreasing autophagosome-lysosome fusion. Autophagy (2018) 14(8):1435–55. doi: 10.1080/15548627.2018.1474314 PMC6103682 29940786

[51] Murray J Thomas H Berry P Kyle S Patterson M Jones C . Tumour cell retention of rucaparib, sustained PARP inhibition and efficacy of weekly as well as daily schedules. Br J Cancer (2014) 110(8):1977–84. doi: 10.1038/bjc.2014.91 PMC3992512 24556618

[52] Mateo J Carreira S Sandhu S Miranda S Mossop H Perez-Lopez R . DNA-Repair defects and olaparib in metastatic prostate cancer. N Engl J Med (2015) 373(18):1697–708. doi: 10.1056/NEJMoa1506859 PMC5228595 26510020

[53] Golan T Hammel P Reni M Van Cutsem E Macarulla T Hall MJ . Maintenance olaparib for germline BRCA-mutated metastatic pancreatic cancer. N Engl J Med (2019) 381(4):317–27. doi: 10.1056/NEJMoa1903387 PMC6810605 31157963

[54] Mateo J Porta N Bianchini D McGovern U Elliott T Jones R . Olaparib in patients with metastatic castration-resistant prostate cancer with DNA repair gene aberrations (TOPARP-b): a multicentre, open-label, randomised, phase 2 trial. Lancet Oncol (2020) 21(1):162–74. doi: 10.1016/S1470-2045(19)30684-9 PMC6941219 31806540

