# Supplementary material for: KP372-1-Induced AKT Hyperactivation Blocks DNA Repair to Synergize With PARP Inhibitor Rucaparib via Inhibiting FOXO3a/GADD45α Pathway
Source: Front Oncol. 2022 Sep 20;12:976292. doi: 10.3389/fonc.2022.976292 (PMC9530825; doi:10.3389/fonc.2022.976292)
Supplement: Supplementary file 1 [file DataSheet_1.pdf]

SUPPLEMENTARY MATERIALS

The file includes:

Supplemental Material and Methods

Fig. S1. PARP1 is elevated in breast adenocarcinoma and lowly expressed in recalcitrant non-small cell lung cancer (NSCLC) and the lethality of PARP inhibitor with KP372-1 in various cancer cell lines.

Fig. S2. PARP inhibitor blocks KP372-1-induced PARP1 hyperactivation and recovers cellular NAD^+ and ATP losses.

Fig. S3. KP372-1 or KP372-1 + PARP inhibitor rucaparib induces cell stress.

Fig. S4. KP372-1 causes Ca^{2+} releasing to promote AKT hyperactivation.

Fig. S5. KP372-1 overcomes PARP inhibitor resistance via inhibiting FOXO3a/GADD45α.

Fig. S6. KP372-1 synergizes with PARP inhibitor rucaparib against MiaPaCa-2 pancreatic orthotopic xenografts.

Supplemental Materials and Methods

Drugs and reagents

KP372-1 was formulated in DMSO for *in vitro* study; for *in vivo* study, KP372-1 powder was suspended in 20% HPβCD (dissolved in sterile PBS) and sonicated 15-30 seconds at 30 °C in Bransonic® ultrasonic bath till dissolution equilibrium was reached, followed by filtering via Nylon syringe filter (0.2 µM pore size, from Fisher Scientific). Rucaparib was formulated in

DMSO for *in vitro* study and in 0.5% methylcellulose solution for *in vivo*. Antibodies used in this study for immunofluorescence and Western blotting were: NQO1 (A180, Santa Cruz, La Jolla, CA), PARP1 (F-2, Santa Cruz), β-actin (C4, Santa Cruz), α-tubulin (B-7, Santa Cruz), PAR (Trevigen, Gaithersburg, MD), Cleaved caspase 7 (D6H1, Cell Signaling), Cleaved PARP1 (ab4830, Abcam), GADD45a (D17E8, Cell Signaling), pAKT^{s473} (D9E, Cell Signaling), AKT (Cell Signaling), LC3A/B (D3U4C, Cell Signaling), H2AX (938CT5.1.1, Cell Signaling), FOXO3a (Abclonal), RAD51 (EPR4030(3), Abcam), p62 (#5114, Cell Signaling) and γH2AX (JBW301, Millipore, Temecula, CA). siRNA against scrambled (sc-37007), *AKT 1/2* (sc-43609), and *FOXO3a* (sc-37887) were obtained from Santa Cruz Biotechnology, siRNA against *GADD45α* (s3993) was obtained from ThermoFisher Scientific. Annexin V assay kit was purchased from BD Biosciences (BD Pharmingen™). Alkaline comet assay kit was from Trevigen (Gaithersburg, MD). Bafilomycin A1 was purchased from Tocris Bioscience (# 1334).

Cell culture

Cells expect MDA-MB-231 were cultured in DMEM containing heat-inactivated 5% fetal bovine serum in a humidified incubator at 37 °C in 5% CO_2. MDA-MB-231 cells were cultured in 5% fetal bovine serum RPMI 1640 at 37 °C in 5% CO_2.

***NQO1* and *PARP1* knocking out by CRISPR-Cas9**

To generate *NQO1* and *PARP1* knock-out cells, 70% confluent cells were plated and incubated overnight. Transfection was done using Lipofectamine 3000 (ThermoFisher Scientific™) according to the manufacturer's instruction. Plasmid DNA concentrations were as follows: 0.5 μg of guide RNA expression vector and 0.5 μg of Cas9 expression vector were placed in a well. For *PARP1* knockout, 1 μg of CRISPR/Cas9 was placed in a well. After transfection, cells were incubated at 37 °C, 5% CO_2 for 2 days, then *NQO1* KO transfected cells were passed onto new

plates with 10 μg/mL puromycin to select positive cells for 14 days, then followed standard single cell clone selection procedure. *PARP1* KO cells were established after directly followed single cell clone selection procedure with GFP guiding.

siRNA transfection

Cells were seeded on 10 cm plate (70-80% confluence) 24 h prior to transfection and grown in serum containing media. On the experiment day, the media were replaced with Opti-MEM™ (ThermoFisher Scientific™), transfection was done using Lipofectamine RNAiMAX (ThermoFisher Scientific™) according to the manufacturer's instruction. Cells then were incubated (37 °C, 5% CO_2) for 48 h and collected for western blot analysis or seeded on plate for subsequent drug treatments.

Cell survival assay

Relative survival assays based on 7-day DNA content assessments were described as previous report (1). In brief, 10,000 cells/well were seeded on 48-well plate 24 h in advance prior to drug treatment. For synergic treatment, cells were pre-exposed to PARP inhibitors 2 h following the other 2 h with KP372-1 and/or dicoumarol. For KP372-1 alone treatment, cells were exposed to KP372-1 for 2 h. Following that procedure, cell media were replaced with fresh media and the plates were incubated at 37 °C in a humidified incubator with 5% CO_2 for 7 days. Then, plates were washed with 1x PBS, added 200 μL H_2O/well, and frozen at -80 °C for 2 h. After thawing the cells, 200 μL/well TNE buffer (50 mM Tris–HCl (pH 7.4), 100 mM NaCl, 0.1 mM EDTA) with 1 μg/mL Hoechst 33258 was added and incubated for 1 h. Cell growth was determined with multilabel plate reader (PerkinElmer®) using excitation and emission filters centered at 360 nm and 460 nm, respectively. Percentage of cell growth = (100x (cell experimental – blank)) : (cell control).

Colony formation assay

750 cells were seeded on 6 cm plate 24 h prior to drug treatments. Drug treatments were followed by cell survival assay. After treatment, cell media were replaced with fresh media and the plates were incubated at 37 °C in a humidified incubator with 5% CO_2 for 7 days. Then, cell colonies were fixed with absolute methanol at room temperature for 30 min and then stained with 0.5% crystal violet solution for 15 min at room temperature, number of cell colonies was counted under microscope.

Western blotting

0.32M cells were seeded on 10 cm plates 24 h in advance prior to treatment. Treatments were performed as in cell survival assay method. For siRNA experiments, cells were transfected with scrambled oligonucleotides or si*AKT 1/2* or si*FOXO3a* using RNAiMAX (Life Technologies) for 48 h, then passed to new plates to perform treatment next day. After treatment, cells were collected at indicated times by centrifugation, and the cell pellet was suspended in lysis buffer (ThermoFisher Scientific™) containing 1 x protease inhibitor cocktail (ThermoFisher Scientific™) and incubated on ice for 30 min. After centrifugation at 1,4000 rpm for 10 min at 4 °C, the supernatant containing total cell extract was collected, and proteins from cell extracts were quantified using the OD 660 nm assay (Pierce). Around 40 μg cell lysates were resolved on SDS-PAGE gel and transferred onto PVDF membrane (Immobilon®-P, Sigma). The membrane was incubated for 1 h at room temperature in blocking buffer (TBS-T with 5% skimmed milk), and then was probed with indicated primary antibodies overnight at 4 °C and then horseradish alkaline phosphatase-conjugated secondary antibody for 1 h at room temperature. Proteins were visualized with Super Signal West Femto Substrate (ThermoFisher, Waltham, MA) and exposure to film.

Comet and immunofluorescence assays

Drug-treated cells were harvested and washed with 1 x PBS, then $1x10^4$ cells were mixed with 0.7% (f.c.) of low-gelling agarose and were layered on comet slides. Slides were then lysed in lysis buffer at 4 °C for 30 min and electrophoresed in alkaline buffer (200 mM NaOH, 1 mM EDTA, pH >13) for 30 min at 0.23 V/cm, followed by washing with cold distilled H_2O and 70% ethanol, and then dried at room temperature overnight. Slides were then stained with SYBR® Gold TE solution and captured using a Leica DM5500 microscope. Comet tail lengths were quantified by NIH Image J. For γH2AX and RAD51 foci, drug-treated cells were immunofluorescence stained and imaged on a Leica DM5500 fluorescent microscope and quantified for foci/nucleus.

Immunohistochemistry staining

Patient samples were collected during surgical resection and were formalin fixed and paraffin embedded (FFPE) and obtained from Indiana University Melvin and Bren Simon Comprehensive Cancer Center Tissue Bank. Sample use was approved by the Committee on Human Research at Indiana University. PARP1 (1:250, sc-8007, Santa Cruz) staining was done with Dako systems and standard incubation times. Stained slides were imaged on a Leica DM5500 fluorescent microscope, images were quantified by NIH ImageJ2.

O_2 consumption rate assay

O_2 consumption rate was measured using Seahorse 96-well plates in conjunction with an XF96 sensor cartridge and XF96 Extracellular Flux Analyzer (Agilent Technologies, DE) according to the manufacturer's instructions. In brief, 35,000 cells per well were seeded 24 h in advance in DMEM media; media was replaced next day with Seahorse media containing glucose (10

mM), glutamine (2 mM) and pyruvate (1 mM). Levels of O_2 consumption were measured under baseline conditions and in the presence of different compounds.

Antitumor efficacy and pharmacokinetics (PK) studies

To establish orthotopic A549 xenograft-bearing mouse model, 1 x 10^6 cells for one mouse were re-suspended with cold 1 x PBS and were intravenously injected into mouse tail vein. For pancreatic MiaPaCa-2 orthotopic model, mice were opened at the spleen site and then 1 x 10^6 cells (for one mouse) were injected into pancreases. 7 days after injections, mice were split into four groups with equal numbers and given intravenous tail injections of vehicle (20% HPβCD) or rucaparib (10 mg/kg) and/or KP372-1 (16 mg/kg) every other day for total 5 injections. Mice weights were monitored every other day during injection period, then monitored once per week until the weight had decreased (A549 xenograft model) or increased (MiaPaCa-2 orthotopic model) more than 30% of original. The maximum tolerable dose was calculated by *in vivo* dose-response experiments.

To collect samples for PK of rucaparib or KP372-1 assay, 1 x 10^6 cells for one mouse were re-suspended with cold 1 x PBS and were intravenously injected into NOD/SCID mouse tail. After the orthotopic NSCLC A549 tumors were established, vehicle (20% HPβCD) or rucaparib (10 mg/kg) and/or KP372-1 (16 mg/kg) were intravenously injected into tails, then mice were sacrificed at indicated time points and samples were collected.

Supplementary Figures

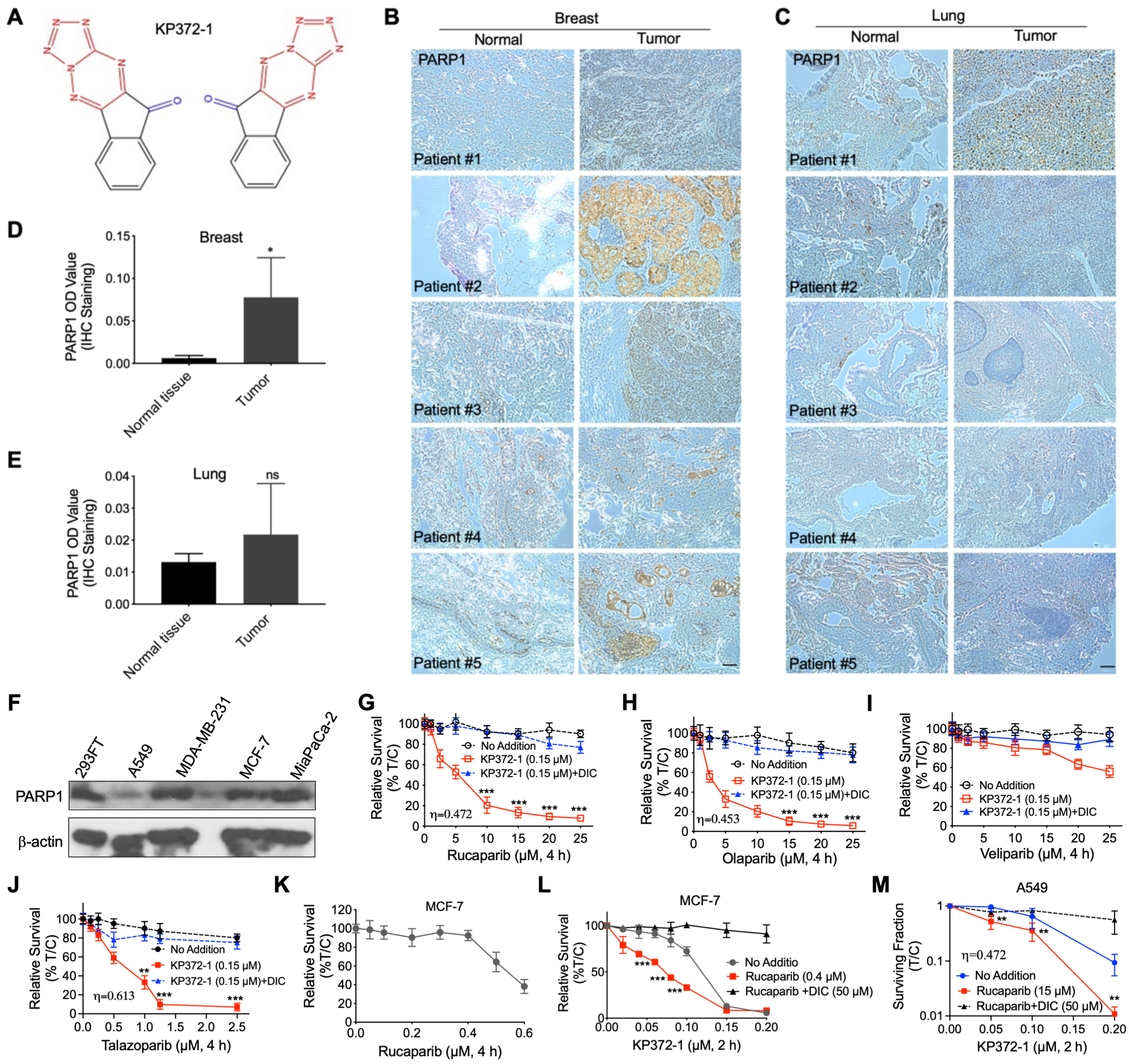

Figure S1. PARP1 is elevated in breast adenocarcinoma and lowly expressed in recalcitrant non-small cell lung cancer (NSCLC) and the lethality of PARP inhibitor with KP372-1 in various cancer cell lines. A, Molecular structure of KP372-1. **B-C**, Immunohistochemical staining of PARP1 in patient samples with breast (**B**) and lung (**C**) adenocarcinomas. **D-E,** Quantification of immunohistochemical staining of PARP1 in breast (**D**) and lung (**E**) adenocarcinomas patient samples. **F,** PARP1 expression in different wild type cell lines. **G-J,** A549 cells were pretreated for 2 h with rucaparib, olaparib, veliparib, and talazoparib, respectively, followed by a 2 h treatment with KP372-1 ± rucaparib, then cells were

washed and replaced with fresh media, and survival assessed after 7 days. Synergy was calculated as per (Chou and Talalay, 1984).(2) Synergy values rucaparib, Olaparib, and talazoparib were reported based on multiple dose-responses, or on comparative p values. **K,** Cell viability of MCF-7 cells treated with different concentration of rucaparib. Cells were treated for 4 h, then followed by washing and replacing media, cell viability was assessed after 7 days. **L,** Survival of MCF-7 cells exposed to KP372-1 ± rucaparib. **M,** Survival fraction of A549 cells treated with KP372-1 ± rucaparib. **L, M**, Cells were pre-treated ± rucaparib for 2 h, then exposed to KP372-1 ± rucaparib for 2 h, followed by washing and replacing fresh media. Cell viability was determined by DNA assay or colony formation assay 7 days later; Data are shown as mean ± SD, each experiment was done three independent times. Scale bar indicates 110 μm. **D-E,** **P* < 0.05, ns: no significant, comparing the PARP1 levels between normal and tumor tissues (*t* tests). **G-M,** **P* < 0.05, ***P* < 0.01, ****P* < 0.001, comparing each data point with those of single treatments (*t* tests).

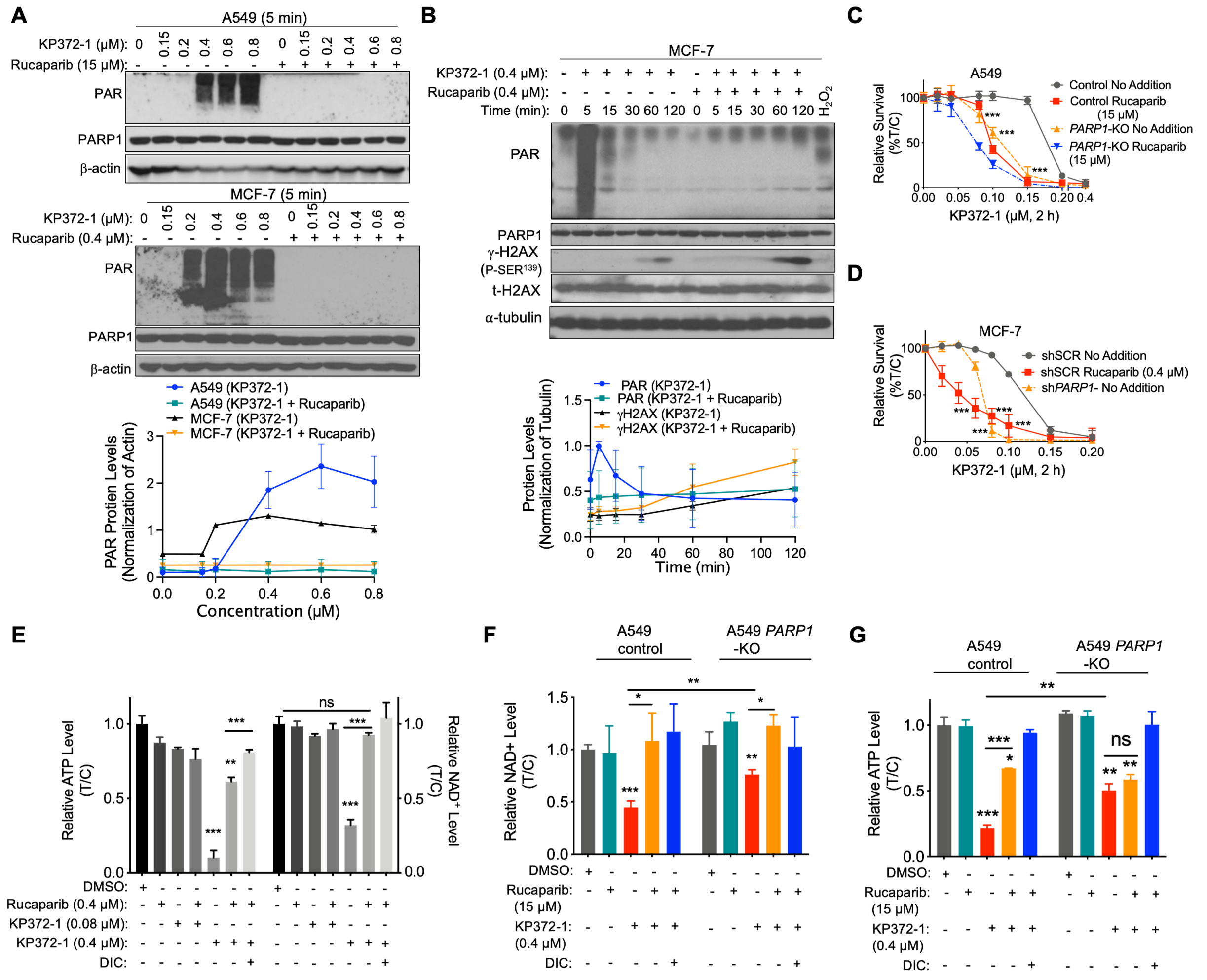

Figure S2. PARP inhibitor blocks KP372-1-induced PARP1 hyperactivation and recovers cellular NAD$^+$ and ATP losses. A, A549 or MCF-7 cells were pre-treated ± rucaparib (15 μM or 0.4 μM, 2 h), then exposed to various dose of KP372-1 ± rucaparib for 5 min, western analysis of PAR formation and PARP1 expression. Bottom panel showed quantification of PAR levels. **B,** MCF-7 cells were pre-treated ± rucaparib (0.4 μM, 2 h), then exposed to KP372-1 (0.4 μM) ± rucaparib for 5 -120 min, PAR and γH2AX formations were assessed via western blot, and bottom panel showed quantification of PAR and γH2AX protein levels. **C-D,** Cell viability of A549 *PARP1* knockout (**C**) and MCF-7 sh*PARP1* cells (**D**) treated with KP372-1 ± rucaparib (pre-treated for 2 h). **E,** MCF-7 cells were pre-treated ± rucaparib (0.4 μM, 2 h), then exposed to KP372-1 (0.15 or 0.4 μM) ± rucaparib for 2 h, relative ATP and NAD$^+$ levels were assessed. **F-G,** A549 and A549 PARP1 knockout cells were pre-treated ± rucaparib (15 μM, 2

h), then exposed to KP372-1 (0.4 μM) ± rucaparib for 2 h, relative NAD^+ (**F**) and ATP (**G**) levels were assessed. All error bars are means ± SD from three independent experiments. **C-D,** ***$P < 0.001$, comparing each data point treatments with KP372-1 treatments in control A549 or shRNA MCF-7 cells (*t* tests). **E-G,** *$P < 0.05$, **$P < 0.01$, ***$P < 0.001$ and ns: no significant, comparing each group with control (DMSO) treatments (*t* tests).

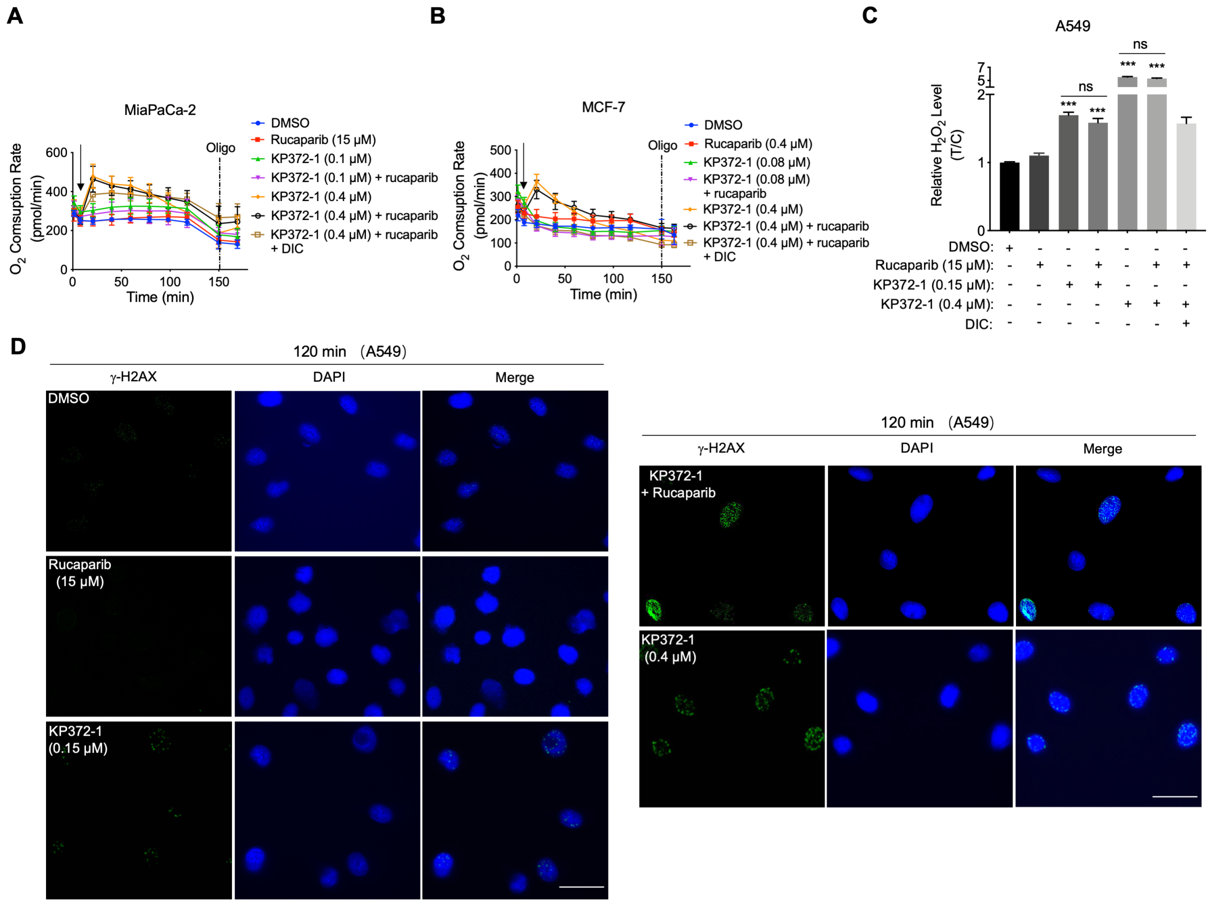

Figure S3. KP372-1 or KP372-1 + PARP inhibitor rucaparib induces cell stress. Cells were pre-treated ± rucaparib (15 µM or 0.4 µM, 2 h), then exposed to KP372-1 (0.1 or 0.4 µM) ± rucaparib for 2 h. Cells were assessed for: **A-B,** Real-time oxygen consumption rates (OCRs) in MiaPaCa-2 (**A**) and MCF-7 (**B**) cells by Seahorse XF analyses. Oligo, oligomycin. **C,** Relative H_2O_2 levels in A549 cells at 2 h. **D.** Full images of immunofluorescence staining of γH2AX in A549 cells. Results were separately repeated at least three times in triplicate each. Scale bar indicates 25 µm. All error bars are means ± SD. **C,** ****P* < 0.001, comparing each group with control (DMSO) treatment (*t* tests), ns: no significant.

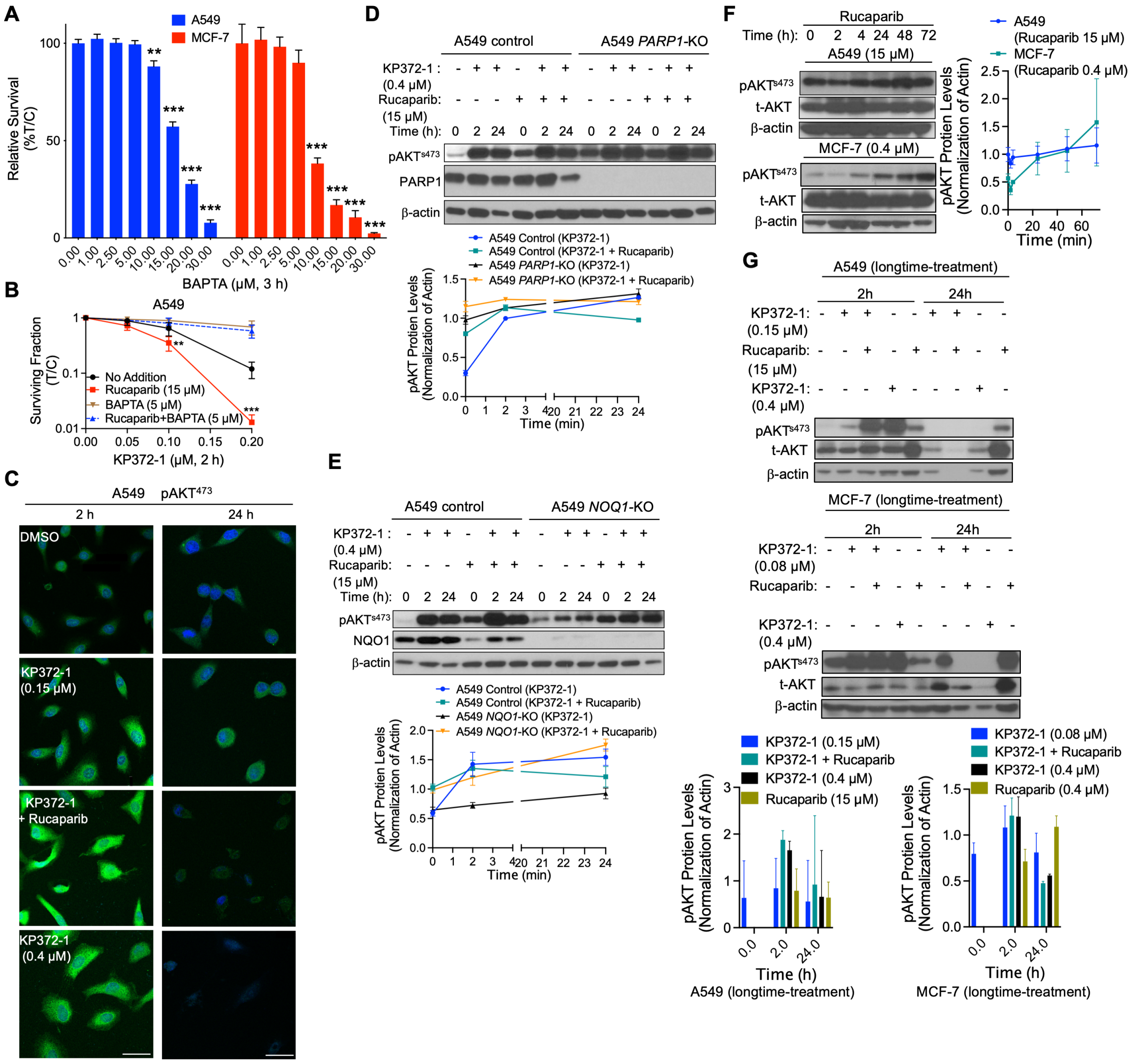

Figure S4. KP372-1 causes Ca^{2+} releasing to promote AKT hyperactivation. A, A549 or MCF-7 cells were exposed to various doses of BAPTA for 3 h, cell viability were assessed by DNA assay. **B,** A549 cells were pre-treated ± rucaparib (15 µM) for 1 h, then added ± BAPTA (5 µM, 1 h), then exposed to KP372-1 ± rucaparib ± BAPTA for 2 h, followed by washing and replacing media, colony formation assay was determined 7 days later. **C,** Full fluorescence images of $pAKT^{s473}$ in A549 cells treated with or without KP372-1 ± rucaparib. **D-E,** A549 *PARP1*-KO or *NQO1*-KO cells were pre-treated ± rucaparib (15 µM) for 2 h, then exposed to KP372-1 ± rucaparib for 2 h, followed by washing and replacing media, cells were collected at indicated time and assessed for: **D,** $pAKT^{s473}$ and PARP1 levels; **E,** $pAKT^{s473}$ and NQO1 levels.

Bottom panels (**D-E**) showed quantification of $pAKT^{s473}$ levels, respectively. **F,** A549 or MCF-7 cells were exposed to rucaparib (15 or 0.4 μM) at indicated time (0-72 h), $pAKT^{s473}$ and total AKT levels were examined by western blot, and right panel showed quantification of $pAKT^{s473}$ levels. **G,** A549 or MCF-7 cells were pre-treated ± rucaparib (15 or 0.4 μM, 2 h), then exposed to KP372-1 (0.15 or 0.08 or 0.4 μM) ± rucaparib for 2 or 24 h, western blot analysis of $pAKT^{s473}$ and total AKT levels. Bottom panels showed quantification of $pAKT^{s473}$ levels. All experiments were performed at least three times. All error bars are means ± SD from three independent experiments. Scale bar indicates 25 μm. **A,** ***$P < 0.001$, comparing each data point with control (0 μM) treatments (*t*-tests). **B,** **$P < 0.01$, and ***$P < 0.001$, comparing each data point with KP372-1 treatments (*t* tests).

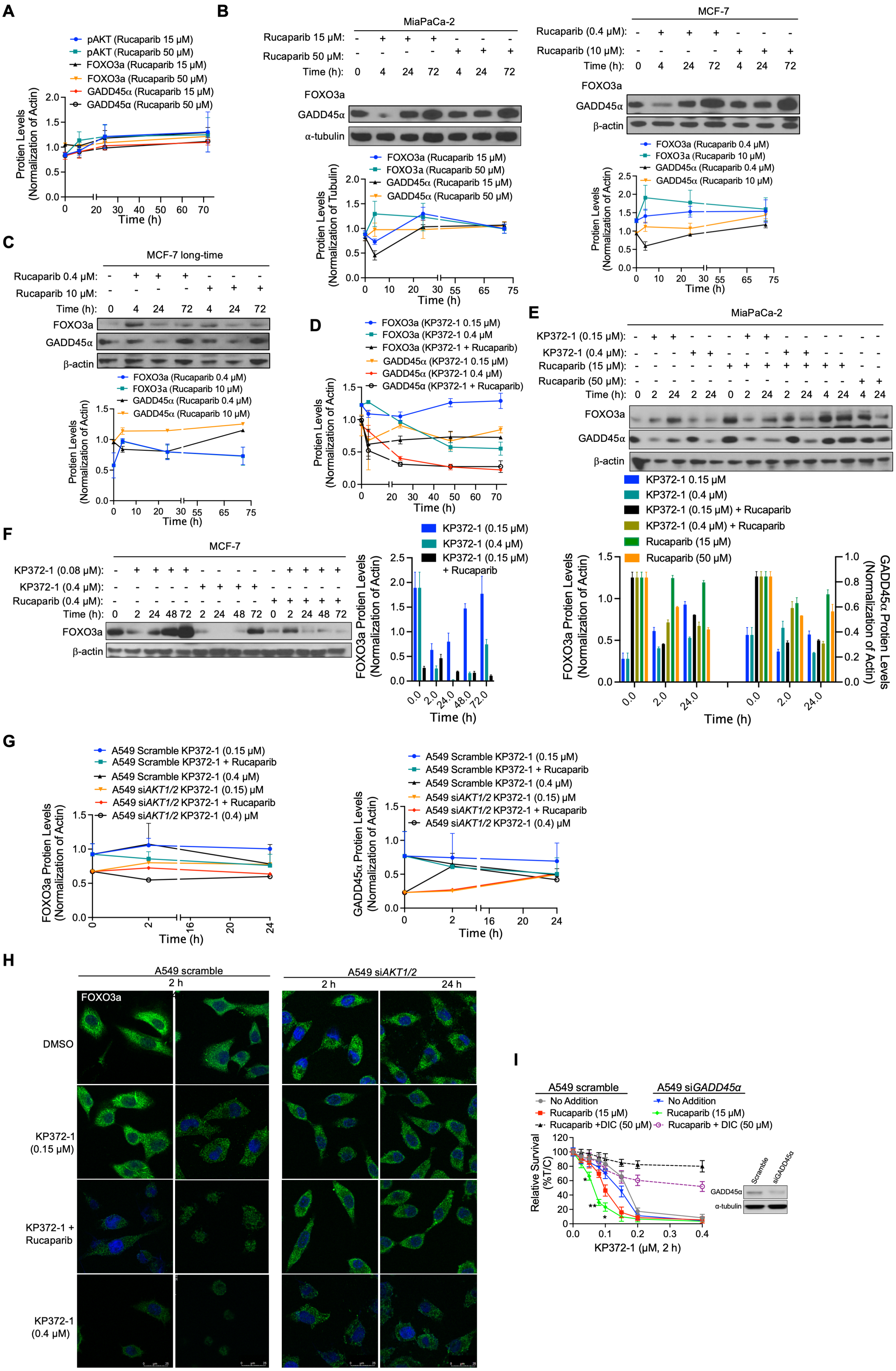
A
pAKT (Rucaparib 15 μM)
pAKT (Rucaparib 50 μM)
FOXO3a (Rucaparib 15 μM)
FOXO3a (Rucaparib 50 μM)
GADD45α (Rucaparib 15 μM)
GADD45α (Rucaparib 50 μM)
Protien Levels (Normalization of Actin)
Time (h)
B
MiaPaCa-2
Rucaparib 15 μM:
Rucaparib 50 μM:
Time (h):
FOXO3a
GADD45α
α-tubulin
FOXO3a (Rucaparib 15 μM)
FOXO3a (Rucaparib 50 μM)
GADD45α (Rucaparib 15 μM)
GADD45α (Rucaparib 50 μM)
Protien Levels (Normalization of Tubulin)
MCF-7
Rucaparib (0.4 μM):
Rucaparib (10 μM):
β-actin
FOXO3a (Rucaparib 0.4 μM)
FOXO3a (Rucaparib 10 μM)
GADD45α (Rucaparib 0.4 μM)
GADD45α (Rucaparib 10 μM)
C
MCF-7 long-time
Rucaparib 0.4 μM:
Rucaparib 10 μM:
D
FOXO3a (KP372-1 0.15 μM)
FOXO3a (KP372-1 0.4 μM)
FOXO3a (KP372-1 + Rucaparib)
GADD45α (KP372-1 0.15 μM)
GADD45α (KP372-1 0.4 μM)
GADD45α (KP372-1 + Rucaparib)
E
MiaPaCa-2
KP372-1 (0.15 μM):
KP372-1 (0.4 μM):
Rucaparib (15 μM):
Rucaparib (50 μM):
KP372-1 0.15 μM
KP372-1 (0.4 μM)
KP372-1 (0.15 μM) + Rucaparib
KP372-1 (0.4 μM) + Rucaparib
Rucaparib (15 μM)
Rucaparib (50 μM)
FOXO3a Protien Levels (Normalization of Actin)
GADD45α Protien Levels (Normalization of Actin)
F
MCF-7
KP372-1 (0.08 μM):
KP372-1 (0.4 μM):
Rucaparib (0.4 μM):
KP372-1 (0.15 μM)
KP372-1 (0.4 μM)
KP372-1 (0.15 μM) + Rucaparib
G
A549 Scramble KP372-1 (0.15 μM)
A549 Scramble KP372-1 + Rucaparib
A549 Scramble KP372-1 (0.4 μM)
A549 siAKT1/2 KP372-1 (0.15) μM
A549 siAKT1/2 KP372-1 + Rucaparib
A549 siAKT1/2 KP372-1 (0.4) μM
H
A549 scramble
A549 siAKT1/2
2 h
24 h
FOXO3a
DMSO
KP372-1 (0.15 μM)
KP372-1 + Rucaparib
KP372-1 (0.4 μM)
I
A549 scramble
A549 siGADD45α
No Addition
Rucaparib (15 μM)
Rucaparib +DIC (50 μM)
Rucaparib + DIC (50 μM)
Relative Survival (%T/C)
KP372-1 (μM, 2 h)
Scramble
siGADD45α
GADD45α
α-tubulin

Figure S5. KP372-1 overcomes PARP inhibitors resistance via inhibiting FOXO3a/GADD45α. A, Proteins quantification of **Fig. 4A**. **B,** MiaPaCa-2 or MCF-7 cells were exposed to rucaparib (15 or 50 μM for MiaPaCa-2, 0.4 or 10 μM for MCF-7) for 4 h, followed by washing and replacing medium, cells were then collected at 4, 24 and 72 h for western blot analysis of FOXO3a and GADD45α, and bottom panels showed quantification of FOXO3a and GADD45α levels. **C,** MCF-7 cells were exposed to rucaparib (0.4 or 10 μM) for 4, 24 or 72 h, then western blot analysis of FOXO3a and GADD45α, and bottom panel showed quantification of FOXO3a and GADD45α levels. **D,** Proteins quantification of **Fig. 4B**. **E-F,** MiaPaCa-2 (**E**) or MCF-7 (**F**) cells were pre-treated ± rucaparib (0.4, 15 or 50 μM, 2 h), then exposed to KP372-1 (0.08, 0.15 or 0.4 μM) ± rucaparib for indicated time (2-72 h), followed by washing and replacing medium, then cells were collected at indicated time, western blot analysis of $pAKT^{s473}$, FOXO3a and GADD45α, and bottom (**E**) and right (**F**) panels showed quantification of FOXO3a and GADD45α levels. **G,** Proteins quantification of **Fig. 4C. H,** *AKT* knockdown A549 cells were pre-treated ± rucaparib (15 μM, 2h), then exposed to KP372-1 ± rucaparib (15 μM) for 2 h followed by washing and replacing media, cells were collected at indicated timepoints and assessed for FOXO3a alterations with full fluorescence image. *AKT* knockdown efficiency was confirmed by Western blot shown in **Fig. 4C**. Scale bar indicates 25 μm. **I,** A549 scramble and si*GADD45α* cells were pre-treated ± rucaparib (15 μM, 2 h), then exposed to KP372-1 ± rucaparib (15 μM) for 2 h followed by washing and replacing media, cell viability was assessed after 7 days. The efficiency of *GADD45α* knockdown was confirmed by western blot analysis. *$P < 0.05$, **$P < 0.01$, comparing each data point of KP372-1 + rucaparib treatment in A549 si*GADD45α* cells with KP372-1 + rucaparib treatment in A549 scramble cells (*t* tests). All experiments were performed at least three times.

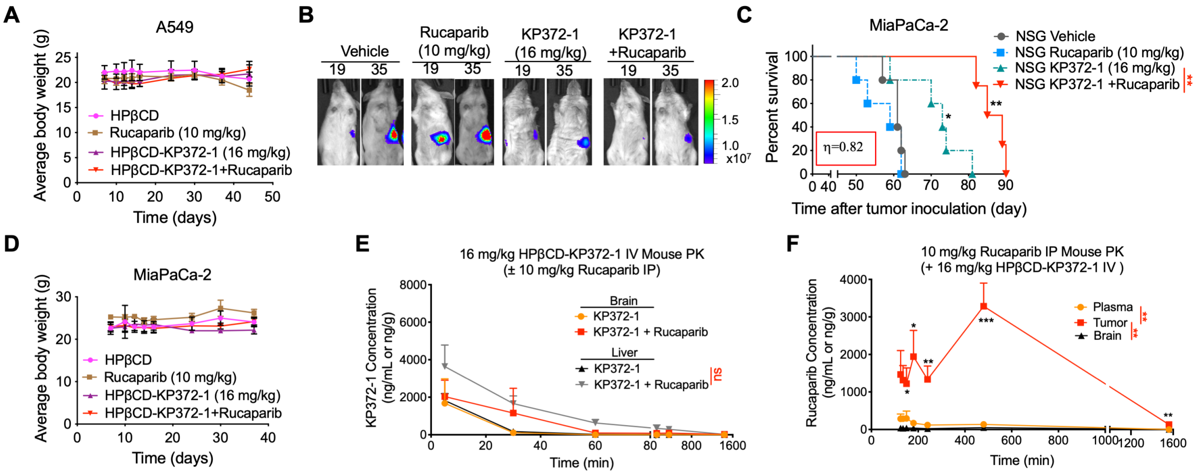

Figure S6. KP372-1 synergizes with PARP inhibitor rucaparib against pancreatic MiaPaCa-2 pancreatic orthotopic xenografts. A, Weight loss of A549 tumour-bearing mice. **B-D,** MiaPaCa-2 orthotopic tumors were established in 20-22 g female NSG mice by injecting 1 x 10^6 MiaPaCa-2 cells into pancreas. After one weeks, mice were treated with or without rucaparib (10 mg/kg, i.p.) for 2 h followed by HPβCD (Vehicle) or HPβCD-KP372-1 (KP372-1) (16 mg/kg, i.v.) every other day for 5 injections via tail vein i.v. Experiments were repeated at least two times. *n* = 5/group. **B,** BLI images of representative mouse tumors at indicated times. **C,** Kaplan-Meier survival curves of MiaPaCa-2 orthotopic mice. $^*P < 0.05$, $^{**}P < 0.01$, KP372-1 (16 mg/kg) or combined treatment vs HPβCD (Vehicle) treatment (log-rank test). Synergy values were reported based on multiple dose-responses (η=0.82). **D,** Weight loss of MiaPaCa-2 tumor-bearing mice. **e** Pharmacokinetics (PK) of KP372-1 in liver and brain tissues of orthotopic A549 tumor-bearing NSG female mice (*n* = 3/group), mice were treated as in **B-D** and sacrificed at indicated times. **F,** Pharmacokinetics (PK) of rucaparib in plasma, tumor and brain of orthotopic A549 tumor-bearing female NSG mice. Data are shown as mean ± SD (**A, D, E, F**), **F,** $^*P < 0.05$, $^{**}P < 0.01$, $^{***}P < 0.001$, comparing each data point with the concentration of rucaparib in plasma (*t* tests), ns: no significant.

Reference:

1. Huang X, Dong Y, Bey EA, Kilgore JA, Bair JS, Li LS, et al. An NQO1 substrate with potent antitumor activity that selectively kills by PARP1-induced programmed necrosis. Cancer Res. 2012;72(12):3038-47.

2. Chou T-C, Talalay P. Quantitative analysis of dose-effect relationships: the combined effects of multiple drugs or enzyme inhibitors. Advances in Enzyme Regulation. 1984;22:27-55.
